# Autophagy mediates proteolysis of NPM1 and HEXIM1 and sensitivity to BET inhibition in AML cells

**DOI:** 10.18632/oncotarget.12493

**Published:** 2016-10-06

**Authors:** Min Huang, Jacqueline S. Garcia, Daniel Thomas, Li Zhu, Le Xuan Truong Nguyen, Steven M. Chan, Ravindra Majeti, Bruno C. Medeiros, Beverly S. Mitchell

**Affiliations:** ^1^ Stanford Cancer Institute, Stanford University, Stanford, California, USA; ^2^ Division of Hematology, Department of Medicine, Stanford University, Stanford, California, USA; ^3^ Institute for Stem Cell Biology and Regenerative Medicine, Stanford University, Stanford, California, USA; ^4^ Department of Pathology, Stanford University School of Medicine, Stanford, California, USA

**Keywords:** autophagy, NPM1, HEXIM1, BET inhibitors, AML

## Abstract

The mechanisms underlying activation of the BET pathway in AML cells remain poorly understood. We have discovered that autophagy is activated in acute leukemia cells expressing mutant nucleophosmin 1 (NPMc+) or MLL-fusion proteins. Autophagy activation results in the degradation of NPM1 and HEXIM1, two negative regulators of BET pathway activation. Inhibition of autophagy with pharmacologic inhibitors or through knocking down autophagy-related gene 5 (Atg5) expression increases the expression of both NPM1 and HEXIM1. The Brd4 inhibitors JQ1 and I-BET-151 also inhibit autophagy and increase NPM1 and HEXIM1 expression. We conclude that the degradation of NPM1 and HEXIM1 through autophagy in certain AML subsets contributes to the activation of the BET pathway in these cells.

## INTRODUCTION

The BET (bromodomain and extraterminal domain) subfamily of bromodomain-containing proteins comprised of Brd2, Brd3, Brd4, and Brdt carry out diverse functions as transcriptional regulators. Of these, Brd4 has emerged as a potentially exciting therapeutic target in a number of malignancies. Brd4 recruits the p-TEFb protein complex that is comprised of cyclin-dependent kinase 9 (Cdk9) and its regulatory partner cyclin T1 to active promoters and super enhancers through its interaction with acetylated histones [1–3]. Through this recruitment, Brd4 positively regulates the transcription elongation carried out by RNA Polymerase II, as well as the expression of a number of other genes involved in oncogenesis including Bcl2 and c-Myc [3–5]. In an opposing interaction, the p-TEFb complex binds to a negative regulatory protein, hexamethylene bisacetamide-inducible protein 1 (HEXIM1) [3–5] that inhibits p-TEFb activity. It is the equilibrium between the positive regulation of pTEFb by Brd4 and its negative regulation by HEXIM1 that determines the extent of activation of the BET pathway [3, 4, 6, 7].

Mutations in the C-terminus of the Nucleophosmin 1 gene (NPMc+) result in the aberrant export of the encoded protein to the cytoplasm [8] and are found in approximately one third of all adult AML, while Mixed Lineage Leukemias (MLL) are characterized by chromosomal translocations involving the fusions of the N-terminus of the MLL gene with many partner genes such as AF4, AF9, ENL or ELL1 [9, 10]. Both the NPMc+ and MLL fusion oncoproteins have been functionally linked to activation of the BET pathway through their interactions with Brd4, HEXIM1, or components of the p-TEFb complex [7, 9–13] and both are associated with activation of the core Brd4 transcriptional program.

Small-molecule inhibitors of BET family proteins have shown promise as antileukemic agents in some leukemia cell lines and primary leukemic cells harboring MLL fusions or NPMc+ mutations [6, 7, 14–17], although resistance has also been documented [16, 17]. It is thus of paramount importance to understand more of the underlying mechanisms that dictate sensitivity or resistance to BET inhibition if these compounds are to be clinically useful. We have discovered that HEXIM1 and NPM1/NPMc+ proteins, key regulators of BET activity, are degraded through the process of autophagy in a number of acute leukemia cell lines and primary cells that express NPMc+ or MLL fusion proteins. Inhibition of autophagy substantially increases the expression of both proteins, as do Brd4 inhibitors such as JQ1 and I-BET151. Our results suggest that the activation of autophagy in these AML cell types confer sensitivity to BET inhibitors, while BET inhibitors appear to also inhibit autophagy.

## RESULTS

### Reduced expression of NPM1 and HEXIM1 in AML cell lines expressing NPMc+ or MLL fusion proteins

**Figure 1:**
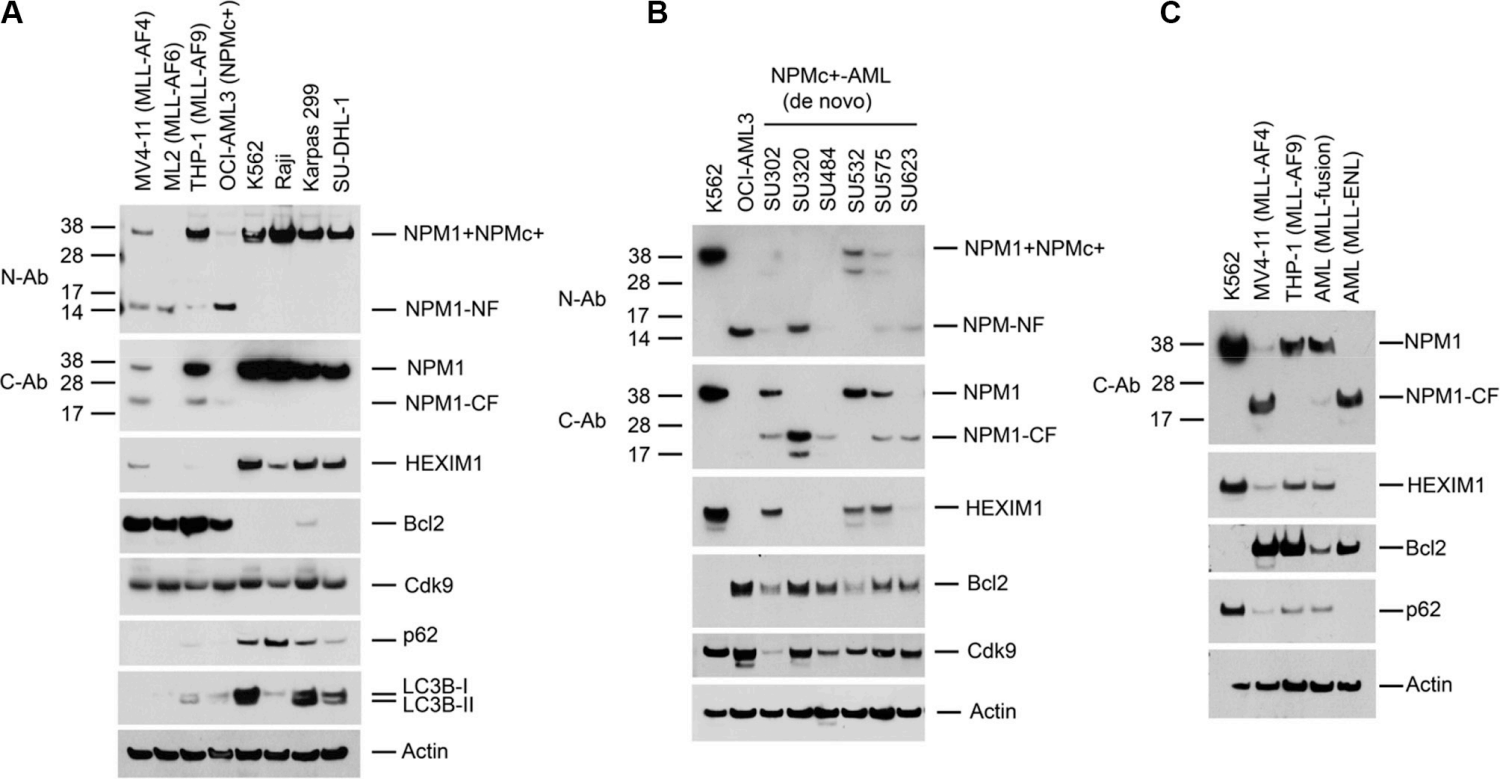
Proteolysis of NPM1 and HEXIM1 in AML cells expressing MLL-fusion proteins or NPMc+ (**A**) Western blot of whole cell lysates from leukemic and lymphoma cell lines. Cell lines: MV4-11 (MLL-AF4), ML2 (MLL-AF6), THP-1 (MLL-AF9), OCI-AML3 cells (NPMc+), K562, Raji, Karpas299, and SU-DHL-1 cells. C-Ab recognizes the C-terminus of wt-NPM1; N-Ab recognizes the N-terminus of both NPM1 and NPMc+. C (CF)- and N (NF)-terminal degradation fragments of NPM1/NPMc+ are indicated. (**B**, **C**) Western blots of lysates of leukemic blasts obtained from six NPMc+ AML patients with de novo disease and two MLL fusion AML patients. The characteristics of the primary AML cells are shown in Supplementary Figure 11.

We examined the expression of NPM1 and HEXIM1 in three cell lines that express MLL fusion proteins and in the OCI-AML3 cell line that expresses NPMc+. Western blots of cell lysates performed with antibodies specific to the C- (C-Ab) or N- (N-Ab) termini of NPM1 revealed fragments of degraded NPM1 protein (CF and NF, respectively) and a reduction in the overall expression of NPM1 as compared with its expression in a variety of other leukemia and lymphoma cell lines (K562, Raji, Karpas 299 and SU-DHL-1) (Figure 1A). The proteolytic cleavage of NPM1 is associated with a marked reduction in expression of HEXIM1 and an increase in Bcl2 expression (Figure 1A). Similar results were obtained using leukemic blasts isolated from five of six patients with de novo NPMc+ AML (SU302, SU320, SU484, SU575, and SU623) (Figure 1B) and two patients with MLL fusion AML (Figure 1C).

The specificity of the N- and C- antibodies for NPM1 is shown in Supplementary Figure S1A. Immunoprecipitation of wt-NPM1 from OCI-AML3 cell lysate using the C-Ab followed by Edman sequencing identified the major NPM1 cleavage site as lying between Lys 134 and Leu 135 (Supplementary Figure S1B and S1C). Cleavage at this site leaves the nuclear localization signal (NLS) intact in the C-terminal fragment of approximately 22 kDa (Supplementary Figure S1D).

### Role of autophagy in protein degradation

**Figure 2:**
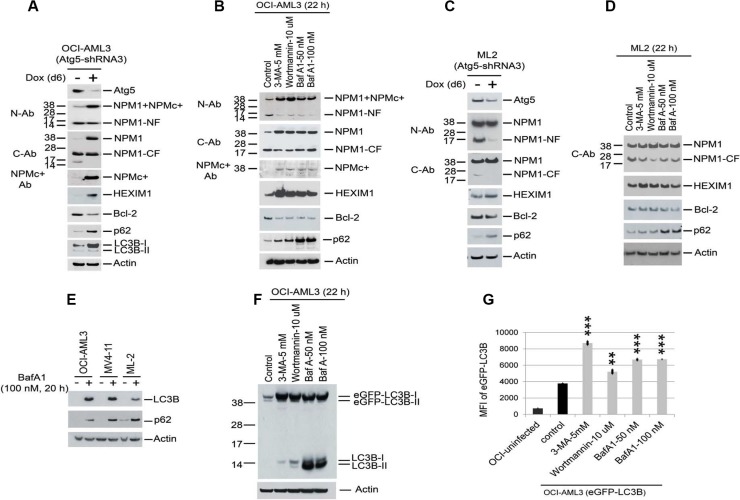
Reduction of proteolytic degradation of NPM1/NPMc+, HEXIM1, and LC3B with autophagy inhibition (**A**, **C**) OCI-AML3 and ML2 cells stably expressing inducible Atg5 shRNA were treated with vehicle or doxycycline for six days to induce Atg5 shRNA expression, followed by Western blot analysis. (**B**, **D**, **E**, **F**) OCI-AML3, MV4-11, ML2 cells, and OCI-AML3 cells stably expressing eGFP-LC3B were incubated with the autophagy inhibitors 3-MA, Wortmannin, or BafA1 at the concentrations shown for 20 h or 22 h followed by Western blot analysis. (**G**) Flow cytometric analysis of eGFP-LC3B fluorescent intensity in cells expressing eGFP-LC3B fusion protein after incubation with 3-MA (5 mM), wortmannin (10 μM), or BafA1 (50 nM, 100 nM) for 24 h. MFI is the mean fluorescent intensity of GFP-LC3B. Graphs represent the mean ± S.D. of biological triplicate. Asterisks (**) and (***) indicate *p* < 0.01 and *p* < 0.001, respectively, in relation to control un-treated cells.

The proteolytic cleavage of NPM1 in conjunction with markedly reduced expression of p62 and LC3B (Figure 1A) suggested the presence of autophagy, a process through which proteins are targeted for degradation in autophagocytic vesicles. To test for the presence of autophagy, we depleted Atg5, an essential component of the autophagy pathway. In addition, cells were incubated with three pharmacologic inhibitors of autophagy, 3-methyladenine (3-MA), Wortmannin, and BafA1 (Figure 2A and 2B). Both approaches reduced the fragmentation of NPM1, increased the protein expression of NPM1, NPMc+, HEXIM1, LC3B, and p62, and decreased the protein expression of Bcl2 in OCI-AML3 cells. Similar, although less pronounced, effects were observed in ML-2 cells (Figure 2C and 2D). We then asked whether the observed increase in NPM1 and HEXIM1 protein corresponded to alterations in transcription by determining the effect of Atg5 depletion on the expression of HEXIM1, NPM1, and Bcl2 at the mRNA level using Q-PCR. NPM1 mRNA expression was unchanged, whereas the expression of Bcl2 and c-Myc, two Brd4 target genes, was significantly reduced in OCI-AML3 and ML2 cells (Supplementary Figure S2A and S2B). Atg5 depletion did not significantly alter HEXIM1 mRNA expression in OCI-AML3 cells (Supplementary Figure S2A) but decreased its expression in ML-2 cells (Supplementary Figure S2B).

In order to quantitatively evaluate the extent of autophagy in OCI-AML3 compared to K562 cells, we transfected both cell lines with the fluorescently-tagged reporter mCherry-eGFP-LC3B to track LC3B turnover. Green fluorescence due to the GFP tag is lost in acidic vacuoles containing lysosomes whereas red fluorescence from mCherry is retained [18, 19]. Confocal microscopic analysis of fixed cells (Supplementary Figure S3) demonstrated that mCherry-eGFP-LC3B was predominantly located in characteristic red punctae that mark both the non-acidic autophagosomes and the acidic autolysosomes or in green puncta-like structures that mark only the non-acidic autophagosomes in OCI-AML3 cells; such punctae were not prominent in K562 cells (Supplementary Figure S3). Treatment with chloroquine (CQ), an inhibitor of late autophagy [20], increased the accumulation of both red and green punctae in the OCI-AML3 cells, whereas 3-MA, an inhibitor of early events in autophagy [20], caused the re-distribution of fluorescence in the cytoplasm in a diffuse pattern. Similar results were obtained with live cell imaging (Supplementary Figure S4). LC3B and p62 protein stability, as measured by Western blotting (Figure 2A–2F, and Supplementary Figure S5C) and the mean fluorescence intensity (MFI) of GFP-LC3B as measured by flow cytometry (Figure 2G), were both significantly increased in the presence of the three autophagy inhibitors and with Atg5 depletion.

The conjugation of phosphatidylethanolamine to LC3-I to form LC3-II is required for autophagosome formation [21] and LC3-II is tightly bound to autophagosomal membranes [22]. Both endogenous LC3B-I and eGFP-LC3B-I were markedly increased in OCI-AML3 cells treated with autophagy inhibitors (Figure 2F) while the conversion of LC3B-I to LC3B-II was reduced in OCI-AML3 cells treated with 3-MA. However, BafA1, as an inhibitor of late autophagy, increased both LC3B-I and LC3B-II levels as expected (Figure 2F and Supplementary Figure S5C).

As additional pieces of evidence for the presence of autophagy, transmission electron microscopy revealed double-membrane autophagic vacuoles in OCI-AML3 cells (Supplementary Figure S5B and inset). The numbers of autophagic vacuoles were increased with CQ-induced inhibition of autophagy and markedly reduced with Atg5-depletion (Supplementary Figure S5A, S5B, and inset). Furthermore, NPM1/NPMc+, as recognized by the NPM1 antibody (generated with a peptide corresponding to amino acids 81-294 of human NPM1) and HEXIM1 also largely co-localized with p62, a marker of autophagy, as shown using confocal microscopy, (Supplementary Figures S6 and S7). Cumulatively, these findings strongly support the activation of basal autophagy and the degradation of NPM1/NPMc+ and HEXIM1 by autophagy in actively proliferating OCI-AML3 cells.

### Reversal of proteolysis and autophagy with Brd4 inhibition

**Figure 3:**
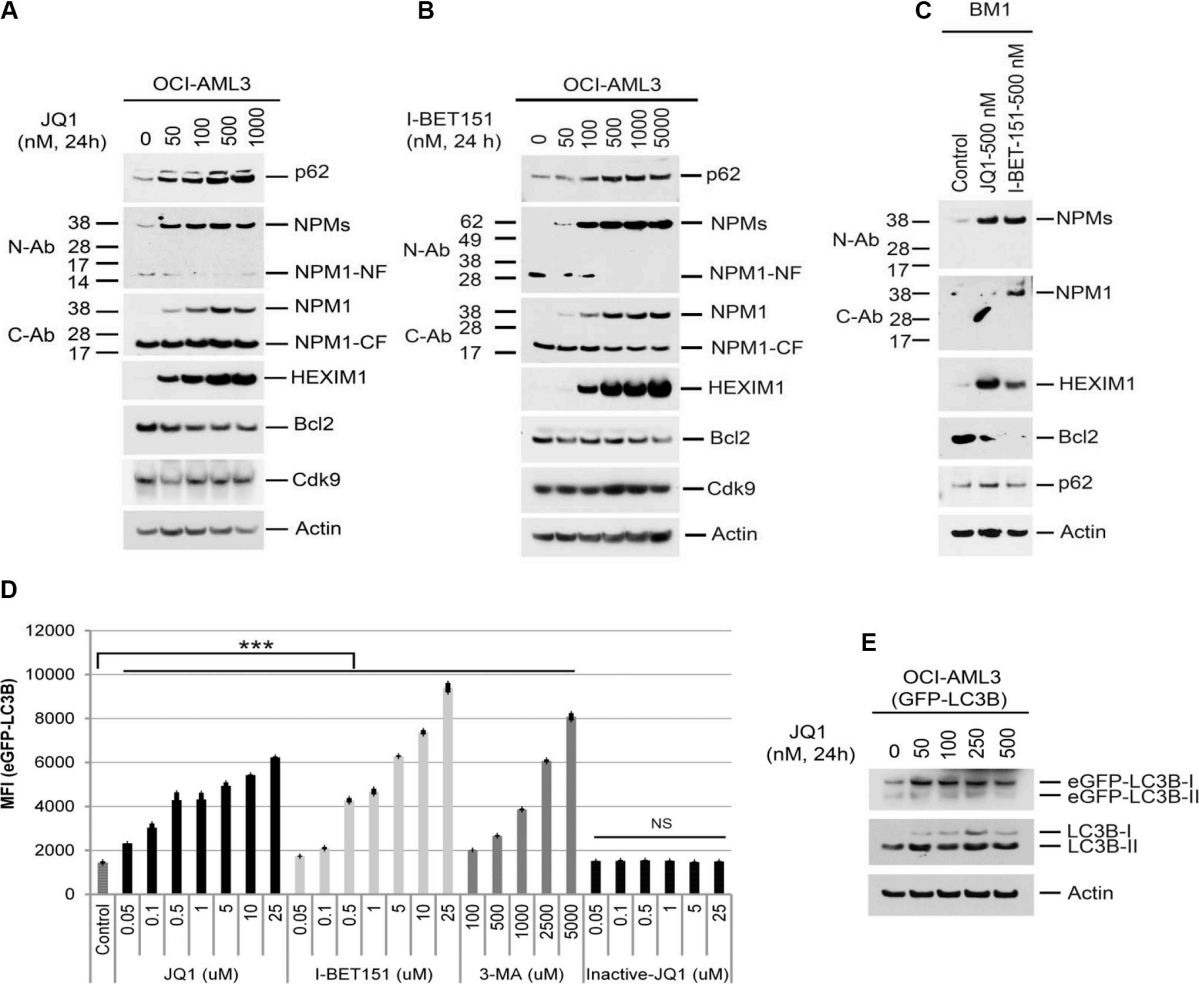
Effects of BET inhibitors on protein expression and autophagy (**A**, **B**, **C**) OCI-AML3 cells or primary NPMc+ AML cells from patient BM1 were treated with I-BET151 or JQ1 at the indicated concentrations for 24 h, followed by Western blot analysis of the proteins shown. (**D**, **E**) OCI-AML3 cells stably expressing eGFP-LC3B were treated with the BET inhibitors JQ1,I-BET151, 3-MA, or (−) JQ1 at the concentrations shown for 24 h, followed by flow cytometric analysis of GFP-LC3B fluorescent intensity (D) or Western Blot analysis (E). Bar graphs in Figure 3D represent the mean ± S.D. of biological triplicate. NS represents non-significance. Asterisks (***) indicate *p* < 0.001 in relation to control un-treated cells.

**Figure 4:**
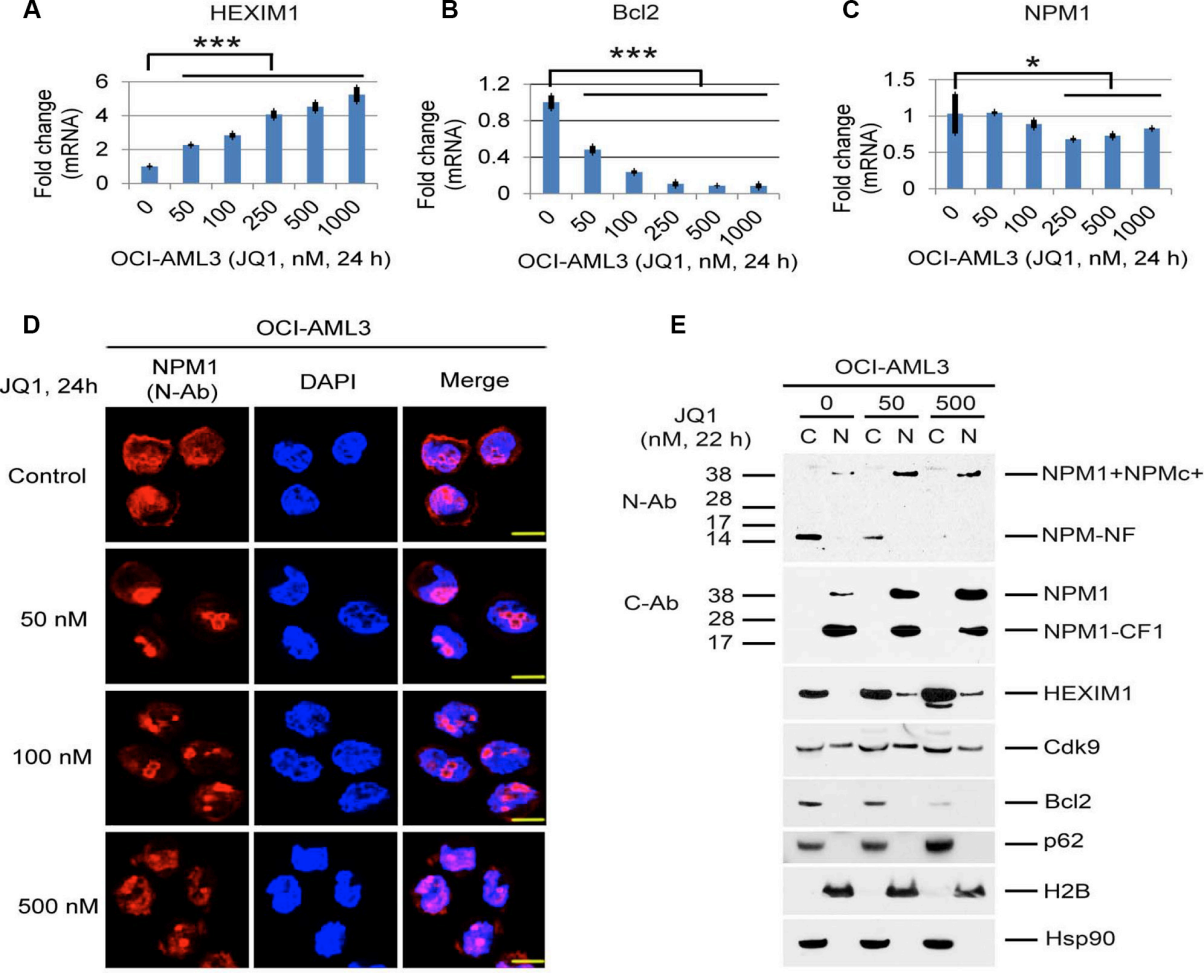
Effects of BET inhibitors on mRNA expression and the cellular distribution of NPM1/NPMc+ and HEXIM1 in OCI-AML3 cells (**A**, **B**, **C**) Effects of JQ1 on the mRNA expression of NPM1, Bcl2, and HEXIM1 in OCI-AML3 cells. OCI-AML3 cells were untreated or treated with JQ1 at the concentrations shown for 24 h, followed by q-PCR analysis. The relative levels of mRNA expression were calculated using the 2^−ΔΔCt^ method after normalization to the GAPDH level and were expressed as fold changes relative to control (set at 1). The mean ± S.D. of four replicates is represented in the bar graphs of Figure 4A, 4B, 4C. Asterisks (*) and (***) indicate *p* < 0.05 and *p* < 0.001, respectively, in relation to control cells. (**D**) Representative immuno-stained images of OCI-AML3 cells untreated or treated with JQ1 at the concentrations shown for 24 h. The N-terminal NPM1 antibody (N-Ab, green) recognizes both wt-NPM1 and NPMc+. The staining for DAPI was shown in blue. Scale bar, 200 px. (**E**) OCI-AML3 cells were untreated or treated with JQ1 at the indicated concentrations for 22 h, followed by cytoplasmic (column C) and nuclear (column N) fractionation and Western blot analysis.

We next investigated the role of the BET pathway in the regulation of autophagy activation. Treatment of OCI-AML3 cells or primary NPMc+ AML blasts (BM1) with JQ1 or I-BET151 substantially increased the expression of NPM1/NPMc+ and HEXIM1 while concomitantly reducing the expression of Bcl2 (Figure 3A, 3B, and 3C). Furthermore, JQ1, I-BET151, and 3-MA all enhanced eGFP-LC3B fluorescence in a dose-dependent fashion (Figure 3D and Supplementary Figure S8). In contrast, (−)-JQ1, the *inactive enantiomer* of JQ1, had no effect on GFP-LC3B protein stability (Figure 3D). An increase in both eGFP-LC3B-I and endogenous LC3B-I was also seen in JQ1-treated OCI-AML3 cells stably expressing GFP-LC3B (Figure 3E). To examine the effects of BET inhibitors on autophagic flux, we performed confocal microscopic live cell imaging analysis. As shown in Supplementary Figure S8D, autophagic punctae are prevalent in OCI-AML3 cells expressing mCherry and GFP dual-tagged LC3B. Treatment of these cells with JQ1 or I-BET151 induced an increase in both GFP and mCherry fluorescence that is diffusely distributed, a pattern similar to that induced by 3-MA, suggesting that BET inhibitors inhibit autophagy flux mainly at an early stage of autophagy. JQ1 also induced the expression of HEXIM1 mRNA while markedly decreasing the Bcl2 mRNA levels, as recently reported [16, 17, 23, 24]. NPM1 mRNA expression was relatively unchanged until JQ1 concentrations exceeded 250 nM (Figure 4A–4C). These results demonstrate the marked similarity of effects of BET inhibitors to those of autophagy inhibitors and support the hypothesis that BET inhibitors increase NPM1/NPMc+ expression primarily through autophagy inhibition.

### Nucleolar and nuclear relocalization of NPM1 and NPMc+ with BET inhibition

Treatment of OCI-AML3 cells with JQ1 also resulted in a marked increase in the nuclear/nucleolar localization of NPM1/NPMc+, as shown by immunostaining using a N-terminal antibody to NPM1 (N-Ab). Cytoplasmic staining was concomitantly diminished (Figure 4D). In contrast, the use of a C-terminal antibody (C-Ab) that recognizes only wt-NPM1 demonstrated that wt-NPM1 remained in the nucleus (Figure 4E). Cellular fractionation studies confirmed that full length NPM1, NPMc+, and the C-terminal cleavage fragments containing the nuclear localization signal (NLS) were primarily in the nuclear fraction while the N-terminal fragment generated from both wt-NPM1 and NPMc+ was mainly in the cytoplasm (Figure 4E). Strikingly, the amount of the N-terminal degradation fragment in the cytoplasm was drastically reduced after treatment with JQ1, whereas the amount of full-length NPM1 and NPMc+ in the nuclear fraction markedly increased. HEXIM1 was predominantly in the cytoplasmic fraction and partially relocated to the nucleus after JQ1 incubation. There were no apparent changes in the cellular distribution of Cdk9, p62, Bcl2, H2B, and Hsp90 after 22 h of JQ1 incubation (Figure 4E). Whether the redistribution of NPMc+ and HEXIM1 out of the cytoplasm by BET inhibitors plays any role in the inhibition of autophagy or in the therapeutic effects of these agents is unclear at the present time.

### Effects of autophagy inhibition and BET inhibition on cell growth

**Figure 5:**
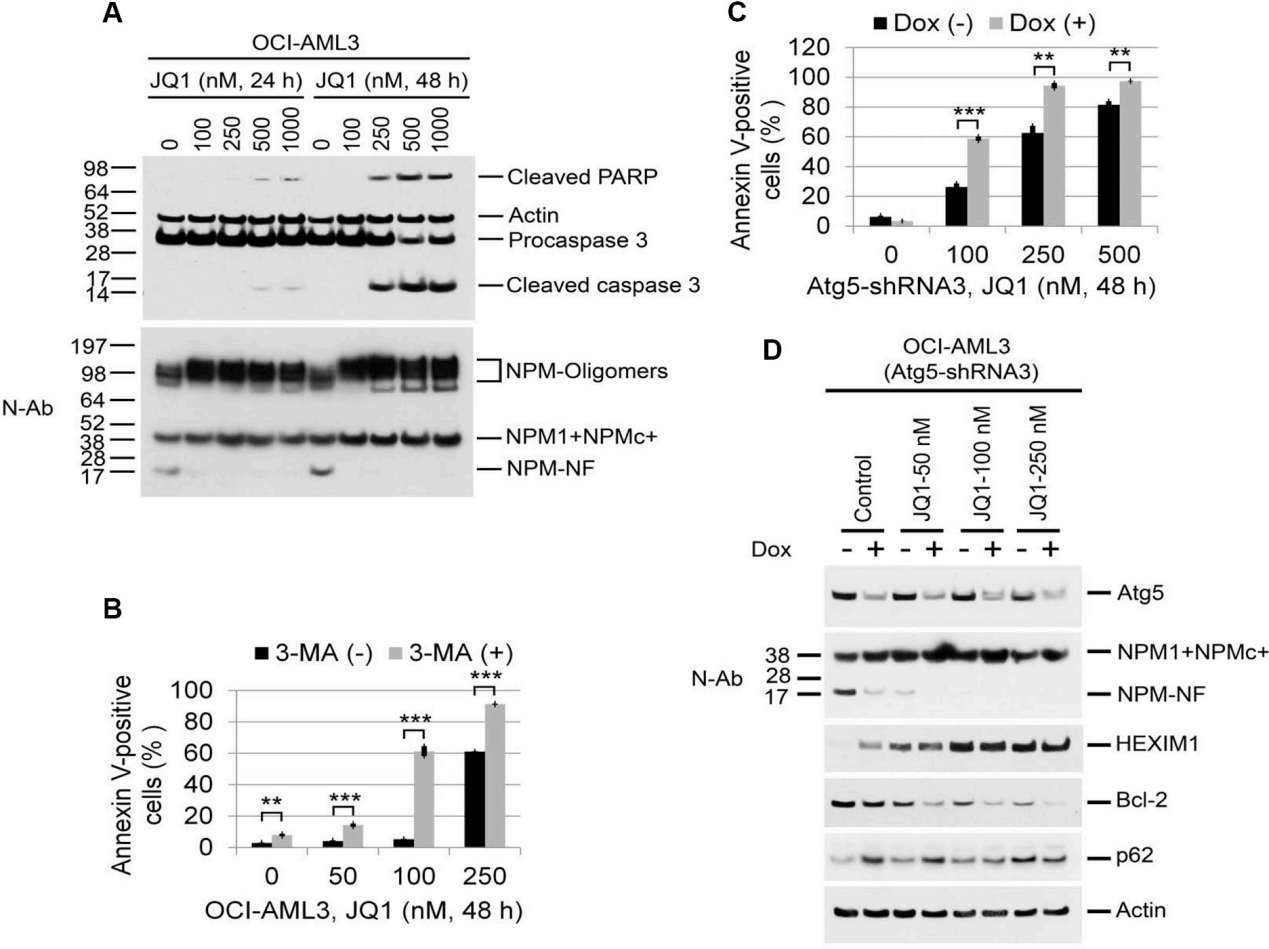
Effects of autophagy inhibition on JQ1-induced apoptosis and protein expression in OCI-AML3 cells (**A**) OCI-AML3 cells were untreated or treated with JQ1 at the indicated concentrations for 24 h or 48 h, followed by Western blot analysis of the proteins shown. Apoptosis Western Blot antibody Cocktail (pro/p17-caspase 3, cleaved-PARP, muscle actin) from Abcam (ab136812) detect apoptosis biomarkers caspase 3 and PARP, along with loading control muscle actin (42 kDa). The caspase 3 antibody detects both the 32 kDa pro-caspase 3 as well as the p17 subunit of active caspase 3 generated by cleavage of the pro-caspase 3 at Asp175. The PARP antibody detects only the apoptosis-specific 89 kDa PARP fragment (cleaved-PARP) generated from the full length PARP by active caspases. (**B**, **C**) Effect of Atg5-shRNA induction and the autophagy inhibitor 3-MA (5 mM) on JQ1-induced apoptosis in OCI-AML3 cells. (B) OCI-AML3 cells were treated with JQ1 or 3-MA alone or in combination at the indicated concentrations for 48 h, followed by flow cytometric analysis of apoptosis. Bar graphs represent the mean ± S.D. of biological triplicate. Asterisks (**) and (***) indicate *p* < 0.01 and *p* < 0.001, respectively, in relation to control cells. (C) OCI-AML3 cells stably expressing inducible Atg5 shRNA3 were incubated with doxycycline to induce Atg5 shRNA for five days, followed by treatment with JQ1 for 48 h and flow cytometric analysis of Annexin V positivity. Bar graphs represent the mean ± S.D. of biological triplicate. Asterisks (**) and (***) indicate *p* < 0.01 and *p* < 0.001, respectively, in relation to cells without doxycycline induction. (**D**) OCI-AML3 cells stably expressing inducible Atg5 shRNA3 were treated with vehicle or doxycycline to induce Atg5 shRNA for five days, followed by treatment with JQ1 at the indicated concentrations for 22 h, followed by Western blot analysis of the proteins shown.

**Figure 6:**
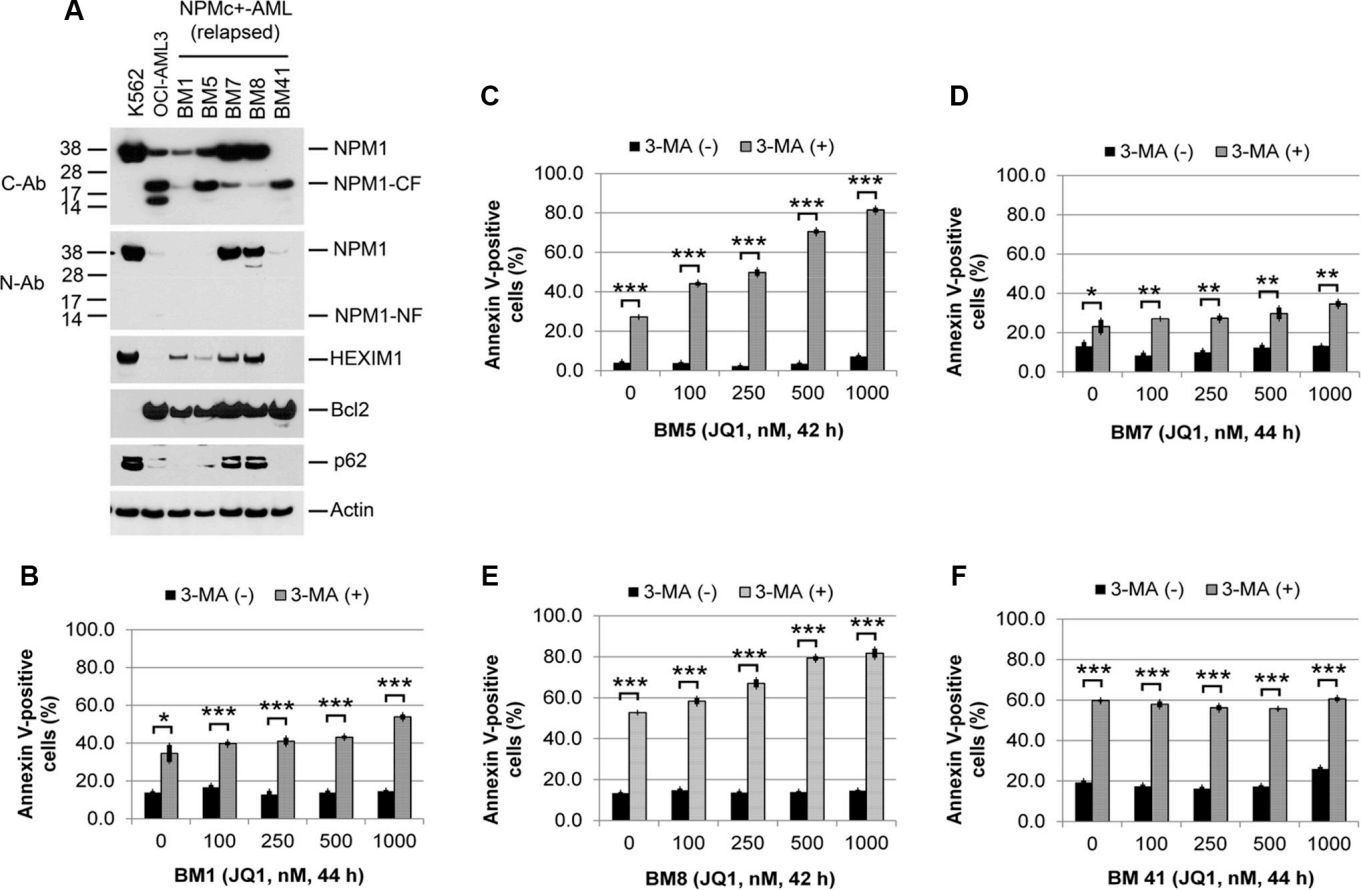
Effects of autophagy inhibition on JQ1-induced apoptosis in primary NPMc+ AML cells (**A**) Western blots of lysates of leukemic blasts obtained from five NPMc+ AML patients with relapsed disease. (**B**, **C**, **D**, **E**, and **F**). Viable mononuclear leukemic cells from each patient were treated with 3-MA (5 mM) or JQ1 alone or in combination at the indicated concentrations and times, followed by flow cytometric analysis of Annexin V positivity. Bar graphs represent mean values ± S.D. of biological triplicate. Asterisks (*), (**), and (***) indicate *p* < 0.05, *p* < 0.01, and *p* < 0.001, respectively, in relation to cells without 3-MA treatment.

JQ1 as a single agent induced apoptotic cell death in OCI-AML3 cells as evidenced by an increased percentage of Annexin V-positively stained cells (Supplementary Figure S9A and S9B) and the induction of both PARP and caspase 3 cleavage fragments in conjunction with reduced procaspase 3 protein expression (Figure 5A). Of additional note in Figure 5A and Supplementary Figure S10A and S14A, high molecular weight oligomers of NPM that are known to be resistant to reducing agents, extensive boiling and SDS denaturation [28–30] are present and also increase as a result of both autophagy inhibition and JQ1 treatment. Depletion of Atg5 or 3-MA treatment alone resulted in minimal growth inhibition or Annexin V positivity in OCI-AML3 cells (Supplementary Figure S10B and S10C), whereas each significantly potentiated the induction of Annexin V positivity by JQ1 (Figure 5B, 5C, and Supplementary Figures S9A, S9B, and S10D). Atg5 depletion combined with JQ1 also increased the expression of NPM1/NPMc+ and HEXIM1 and decreased the expression of Bcl2 to a greater extent than either treatment alone (Figure 5D and Supplementary Figure S10E). To extend these observations to primary AML samples, leukemic blasts from five patients with relapsed or refractory NPMc+ AML (BM1, 5, 7, 8, 41; Supplementary Figure S11) were analyzed for levels of protein expression and sensitivity to JQ1 in the absence or presence of 3-MA (Figure 6, Supplementary Figures S12, S13). Each of these samples displayed some degree of baseline proteolysis and each was relatively resistant to increasing doses of JQ1. However, the addition of 3-MA to JQ1 significantly enhanced Annexin V positivity over the effect of 3-MA alone in three out of five AML samples (BM1, BM5, BM8). This variable response to JQ1 in samples obtained from patients at relapse may be attributable to the acquisition of new mutations or activation of signaling pathways such as the Wnt-beta-catenin pathway that have been implicated as mechanism for resistance to BET inhibitors [16, 17].

**Figure 7:**
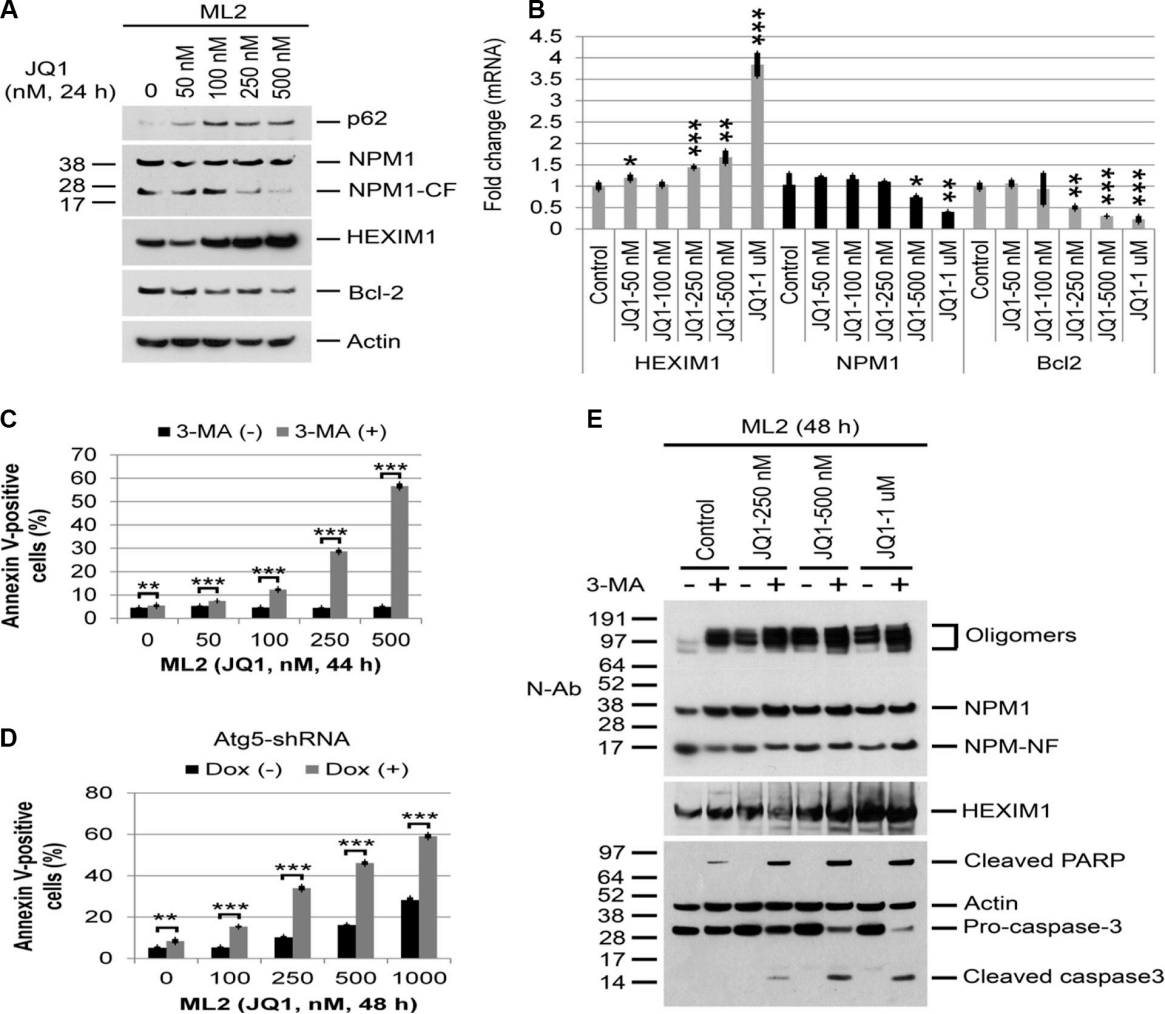
Effects of BET and autophagy inhibition on protein or mRNA expression and Annexin V positivity in MLL cell lines (**A**, **B**) ML2 cells were treated with JQ1 at the indicated concentrations for 24 h, followed by Western blot analysis of the proteins shown or q-PCR analysis of HEXIM1, NPM1, and Bcl2. Bar graphs represent mean values ± S.D. of four replicates. Asterisks (*), (**), and (***) indicate *p* < 0.05, *p* < 0.01, and *p* < 0.001, respectively, in relation to control cells. (**C**) ML2 cells were treated with JQ1 or 3-MA alone or in combination at the indicated concentrations and times, followed by flow cytometric analysis of Annexin V positivity. Bar graphs represent mean values ± S.D. of biological triplicate. Asterisks (**) and (***) indicate *p* < 0.01 and *p* < 0.001, respectively, in relation to cells without 3-MA treatment. (**D**) Effects of Atg5 depletion on JQ1-induced apoptosis in ML2 cells. ML2 cells stably expressing Atg5 shRNA3 were treated with vehicle or doxycycline to induce Atg5 shRNA for five days, then treated with JQ1 for 48 h, followed by flow cytometric analysis of apoptosis. The bar graphs represent mean values ± S.D. of biological triplicate. Asterisks (**) and (***) indicate *p* < 0.01 and *p* < 0.001, respectively, in relation to cells without doxycycline induction. (**E**) ML2 cells were treated with JQ1 or 3-MA (5 mM) alone or in combination at the concentrations shown for 48 h, followed by Western Blot analysis of proteins shown.

Similar experiments were carried out in the MLL cell lines ML2 and THP-1. As with OCI-AML3 cells, treatment with JQ1 increased the protein expression of NPM1, HEXIM1, and p62 in these cells (Figure 7A, Supplementary Figure S14A, S14B, and S14C). JQ1 also induced the expression of HEXIM1 mRNA, markedly decreased Bcl2 mRNA levels, and did not alter NPM1 mRNA levels until concentrations exceeded 500 nM (Figure 7B). The sensitivity of the two MLL cell lines to JQ1 as well as the cleavage of PARP and caspase 3 were also significantly enhanced with 3-MA treatment (Figures 7C, 7E, Supplementary Figures S14D, S15B) and with Atg5 depletion (Figure 7D, Supplementary Figures S14B, S15A).

## DISCUSSION

Autophagy is a catabolic pathway that directs excess or damaged cytoplasmic constituents to lysosomes for degradation and recycling for anabolic processes. Although autophagy has been implicated in various events related to cancer initiation and progression, there are limited data regarding its role in hematologic malignances [25, 26]. Autophagy has been shown to participate in the stress response and maintenance of hematopoietic stem and progenitor cells [27, 28], while the loss of two autophagy genes (Atg5, 7) has been shown to contribute to a pre-leukemic phenotype in mice [26, 29, 30]. In addition, autophagy has been shown result in the degradation of the PML/RARA and Bcr-Abl oncoproteins following treatment with arsenic trioxide or all-trans retinoic acid [31–33], putatively through the induction of reactive oxygen species (ROS). Our data strongly support the activation of autophagy as the cause of degradation of NPM1, NPMc+, and HEXIM1 in a subset of acute myelogenous leukemias. Its role in the regulation of NPM and HEXIM1 protein stability was confirmed by the reversal of proteolysis, the decrease in LC3B degradation, and the increase in p62, a key receptor of cargo uptake into autophagosome vesicle, through autophagy inhibition in both cell lines and primary AML cells. In addition, both NPM1 and HEXIM1 co-localize with the autophagy marker p62 to a large extent. Whether the autophagic degradation of NPM and HEXIM1 is initiated directly through association with p62 or indirectly through association with other autophagy substrate proteins remains unknown.

HEXIM1 and NPM1 have both been shown to inhibit the pTEFb complex; HEXIM1 inhibits pTEFb kinase activity by sequestering it in a complex with 7SK snRNA, while NPM1 sequesters Brd4 through a direct protein-protein interaction [7]. From these data, we postulated that AML cells with reduced levels of NPM1/NPMc+ and HEXIM1 might have increased Brd4 activation and therefore enhanced pTEFb-mediated Pol II transcription. Indeed, cells expressing NPMc+ and MLL fusion proteins have been shown to have activation of the Brd4 core transcriptional program [8–10, 16, 17] and both NPMc+ and MLL cells are sensitive to BET inhibition *in vitro* [6, 7, 17]. HEXIM1 also directly interacts with NPM1 and co-translocates with NPMc+ into the cytoplasm [11]. The presence of HEXIM1 in the cytoplasm in association with NPMc+ may increase the accessibility of both proteins to autophagic lysosomes and possibly account for the more pronounced effects of autophagy inhibition in NPMc+ expressing cells as compared to cells expressing MLL fusion proteins.

We propose a model in which the degradation of NPM1 and HEXIM1 through autophagy diminishes their inhibitory effects on the p-TEFb complex, resulting in increased expression of Brd4-target genes. As has been shown by others, the BET inhibitors JQ1 and I-BET151 are effective inducers of apoptosis in AML cell lines. In the present study, inhibitors of autophagy significantly enhanced apoptosis induced by BET inhibitors, providing some evidence for the intersection of these two pathways (Figures 5, 6, 7). What was unexpected, however, was the ability of BET inhibitors alone to suppress autophagy as shown by their ability to reverse the degradation of HEXIM1, NPM1/c+, LC3B, and p62 (Figures 3, 4, 5, 7), effects very similar to those of known autophagy inhibitors. These data suggest that BET activation itself may play a role in inducing or maintaining the autophagic process. We therefore propose a feedback loop in which autophagy, activated through the expression of the NPMc+ and MLL oncoproteins, leads to proteolysis of HEXIM1 and NPM1/c+ and the subsequent activation of Brd4, which in turn contributes to ongoing autophagy activation (Supplementary Figure S16). The inhibition of autophagic processes by Brd4 inhibitors is a novel concept that may have important consequences for the use of BET inhibitors in the clinic.

In line with our finding of autophagy in AML cells, a recent report detailing the human autophagy interaction network across cancer types demonstrated increased autophagy-associate mRNA levels in several subtypes of AML including NPM1-mutated, MLL-fusion and CBFB-MYH11-fusion leukemias [34]. High basal levels of autophagy have also been reported in several other cancer types such as melanoma and CNS tumors with BRAFV600E mutations [35–38]. Recent studies have established a link between oncoprotein-induced ROS and the induction of autophagy [39–41] and an increase in ROS production has been demonstrated in AML cells with FLT3-ITD and RAS mutations [39, 42–44]. It is not unlikely that the basal autophagy in NPMc+ and MLL fusion AMLs is, at least in part, due to oncogenic activation of ROS.

Partial proteolytic cleavage of NPM1/NPMc+ results in the generation of two major truncated fragments of NPM1, one from the N-terminus (NPM1-NF1: 1-134 AA) that lacks the nuclear and nucleolar localization domain (NLS), and another from the C-terminus (NPM1-CF1: 135-294 AA) that lacks the N-terminal oligomerization domain (Figures 1–7 and Supplementary Figure S1D). Similar partial protein fragmentation resulting from autophagy has been demonstrated for other proteins, including α-synuclein, tau, and histones [45–54]. Potential mechanisms that have been proposed include inefficient activity of lysosomal proteases and partial lysosomal membrane permeabilization, resulting in the release of proteolytic enzymes into the cytosol [47, 48]. In the case of NPM1, partial proteolysis results in a N-terminal fragment (NPM1-NF) and a C-terminal fragment (NPM-CF), with greater restoration of the NPM1-NF following autophagy and BET inhibition (Figure 2A–2D; Figure 3A and 3B). Due to its NLS and high affinity for histones [55], The C-terminal fragment of wt-NPM1 is present in the nucleus/nucleolus where it may be protected from autophagy, whereas the N-terminal fragment remains in the cytoplasm. JQ1treatment results in the nuclear translocation of NPM1/NPMc+ and an increase in the nuclear fraction of full-length NPM1/NPMc+ protein as probed with the N-terminal antibody (Figure 4E, top panels). This result raises the possibility that JQ1 may regulate NPM1 and NPMc+ trafficking through an as yet undetermined mechanism.

In summary, our results suggest the existence of a regulatory loop between autophagy-mediated degradation of NPM1 and HEXIM1 and BET activation. Autophagy activation and the subsequent proteolysis of NPM1 and HEXIM1 result in a shift in the balance between the inactive pTEFb-HEXIM1 complex and an active Brd4-pTEFb complex toward the increased expression of Brd4-dependent genes such as Bcl2. This study also provides a potential explanation for the sensitivity of NPMc+ and MLL fusion expressing leukemic cells to BET inhibitors and suggests that the addition of autophagy inhibitors to BET inhibitors may be more effective than either approach alone in the treatment of certain types of AML. Although a number of studies have demonstrated that autophagy may sensitize certain tumor cells to chemotherapeutic agents [35–38], the clinical applications of autophagy inhibitors such as CQ and hydroxychloroquine have been limited by their efficacy and side effects [56, 57]. The potential of combining BET inhibition with autophagy inhibition *in vivo* will have to await the emergence of more clinically efficacious autophagy inhibitors in the future.

## MATERIALS AND METHODS

### Culture of primary AML cells and cell lines

Ficoll-purified mononuclear cells from the bone marrow or peripheral blood of AML patients were obtained after informed consent according to institutional guidelines (Stanford University Institutional Review Board No. 6453). Ficoll-purified mononuclear cells were cultured in modified culture medium consisting of equal parts of EGM-2 complete medium (Lonza, Cologne) and SFEM complete medium (Stem Cell Technology). The K562 leukemia cell line, AML cell lines (MV4-11, ML-2, THP-1), and Burkitt's B-cell lymphoma cells (Raji) were obtained from American Type Culture Collection, the OCI-AML3 cell line from the German Collection of Microorganisms and Cell Cultures, and ALK^+^ anaplastic large cell lymphoma (Karpas-299) and diffuse large B-cell lymphoma (SU-DHL-1) lines from the Cross Cancer Institute, Canada. K562 cells were cultured in Dulbecco's modified Eagle medium (DMEM) supplemented with 10% heat-inactivated fetal bovine serum (FBS), 2 mM glutamine, 100 U ml^−1^ penicillin and 100 μg/ml streptomycin. Raji, Karpas-299, and SU-DHL-1 were culture in RPMI 1640 supplemented with 10% heat-inactivated fetal bovine serum (FBS), 2 mM glutamine, 100 U ml^−1^ penicillin and 100 μg/ml streptomycin. The OCI-AML3 cell line was maintained in MEM-alpha medium supplemented with 20% heat-inactivated fetal bovine serum (FBS), 2 mM glutamine, 100 U ml^−1^ penicillin and 100 μg/ml streptomycin. All experiments were initiated at a cell density of 1 × 10^5^ to 4× 10^5^ cells/ml.

### Retro- and lentiviral vector constructs and establishment of stable cell lines

Retroviral constructs of pBABE-puro mCherry-enhanced green fluorescent protein (EGFP)-LC3B (mCherry-GFP-LC3B, plasmid 22418) and pBABE-puro GFP-LC3 (plasmid 22405) were generated by Jayanta Debnath [58] (Addgene). OCI-AML3 cells and K562 cells were infected with GFP-LC3 or mCherry-GFP-LC3B retrovirus as described previously [59], followed by sorting of GFP- or mCherry-positive cells using a BD Aria II cell sorter (BD Biosciences). The OCI-AML3 cell line containing a doxycycline-inducible NPM1-shRNA lentiviral construct [59] was used to express NPM1-shRNA. The doxycycline-inducible Atg5-shRNA lentiviral constructs were purchased from Open Biosystems (Huntsville, AL, USA; clone ID V2THS_249282 and V3THS_301130). The mature antisense sequences of Atg5-shRNA1 and Atg5-shRNA5 are AAGTTTCTGAGATTGTATG and ATCTCACTAATGTCTTCTT, respectively. OCI-AML3, ML2, and MV4-11 cells were infected with Atg5-shRNA as described [59], sorted for YFP fluorescent after overnight treatment with doxycycline, and cultured in doxycycline-free medium for three weeks.

### Western blot, immunoprecipitation, and edman sequencing

Cell lysis, western blot, and immunoprecipitation were performed as described previously [59, 60]. Cell lysates (1 mg) from OCI-AML3 were incubated first with anti-NPM1 mouse monoclonal antibody for 4 h at 4°C, then with 40 μl of protein A and G agarose beads for 40 min. After five washes with lysis buffer and two washes with ice cold phosphate buffered saline, the anti-NPM1 immunoprecipitated proteins were separated by SDS-PAGE gel, transferred into PVDF membrane, and stained with Ponceau S followed by *Edman* de novo *N-terminal* protein *sequence* analysis at Stanford PAN facility. Antibodies used for Immunoblots and immunostaining: anti-NPM1 rabbit antibody (ab24412, Abcam), anti-LC3B (#2775, Cell Signaling), anti-NPM1 mouse monoclonal antibody (ab10530, Abcam), anti-NPM1 mouse monoclonal antibody (ab40696, Abcam), anti-mutant nucleophosmin polyclonal antibody (PAI-46356, Thermo Scientific), anti-p62 (#5114, Cell Signaling), anti-HEXIM1 (sc-398479, Santa Cruz Biotechnology), anti-Bcl2 (sc-56015, Abcam, anti-actin (A5441, Sigma), and anti-Atg5 (ab10837, Abcam).

### Flow cytometric analyses

Flow cytometric analysis was performed as described previously [61].

### Live cell imaging and immunocytochemistry with confocal microscopy

OCI-AML3 cells or primary AML3 cells stably infected with mCherry-GFP-LC3B were grown in FluoroDish^™^. The live cell images were performed using a Leica Sp8 White Light Confocal (WLL) microscope with extended focus (Leica Microsystems CMS GmbH, Mannheim, Germany) at the Stanford Microscopy Core Facility. OCI-AML3 or K562 cells stably expressing mCherry-GFP-LC3B were fixed with 4% paraformaldehyde in PBS for 20 min, permeabilized with 0.1% Triton X-100 for 15 min, stained with 4′,6-diamidino-2-phenylindole (DAPI) for 5 min and examined using a Leica SP8 confocal system. Immunostaining of NPM1, HEXIM1, and p62 was performed as described previously [59].

### Electron microscopy

Cells (10 × 10^6^ per condition) were pelleted for 5 minutes at RT at 300 × g and fixed in Karnovsky's fixative, followed by standard TEM ultrastructural analyses using JEOL JEM-1400 at 120kV by Stanford Electron Microscopy Core Facility. Photographs were taken using a Gatan Orius digital camera (NIH grant # 1S10RR02678001).

### Nuclear cytoplasmic fractionation

Cytoplasmic and nuclear fractionations were performed using CelLytic NuCLEAR extraction kit from Sigma (Sigma-Aldrich, St. Louis, MO, USA) with a few modifications. Cells were washed two times with PBS and 5 times of packed cell volume (PCV) of 1× hypotonic lysis buffer (10 mM HEPES pH 7.9, 1.5 mM MgCl2, 10 mM KCl) containing mixtures of the protease inhibitors (150 μM Na_3_VO_4_, 0.25 mM PMSF, 5 μg/ml leupeptin, and 10 nM microcystin LR) was added to the cell pellets, followed by isolation of the cytoplasmic fraction according to the CelLytic NuCLEAR extraction kit protocol from Sigma-Aldrich. For extraction of nuclear fractions, after washing twice with 1× hypotonic lysis buffer with the protease inhibitor, the nucleus was first lysed in equal PCV of the cell lysis buffer with the protease inhibitors, followed by sonication to breakdown the nuclei. The cytoplasmic and nuclear fractions were then collected after centrifugation at 10, 000 g for 10 min. Equal amounts of protein from the cytoplasmic fractions were loaded for subsequent Western blot analysis. The nuclear fraction was loaded at 1:1 ratio to the volume of the quantitated cytoplasmic fraction.

### RNA isolation and Real-time RT-PCR analysis

Real-time RT-PCR (q-PCR) analysis was performed using the SYBR Green PCR Master Mix (Applied Biosystems, Life Technologies) and an Applied Biosystems 7900HT instrument (Applied Biosystems, Foster, CA). The specific primers used are listed in the Supplementary Table S1. All quantitative real-time RT-PCRs were performed in four replicates. Gene regulation was quantitated by the 2^−ΔΔCt^ method with normalization to the GAPDH level.

### Statistical analysis

Results are expressed as mean value ± S.D. Significance levels were determined using the Student's *t*-test. *p < 0.05; **p < 0.01; ***p < 0.001.

## SUPPLEMENTARY MATERIALS FIGURES AND TABLES


