# Supplementary material for: Autophagy mediates proteolysis of NPM1 and HEXIM1 and sensitivity to BET inhibition in AML cells
Source: Oncotarget. 2016 Oct 6;7(46):74917–30. doi: 10.18632/oncotarget.12493 (PMC5342712; doi:10.18632/oncotarget.12493)
Supplement: Supplementary file 1 [file oncotarget-07-74917-s001.pdf]

Autophagy mediates proteolysis of NPM1 and HEXIM1 and sensitivity to BET inhibition in AML cells

Supplementary Materials

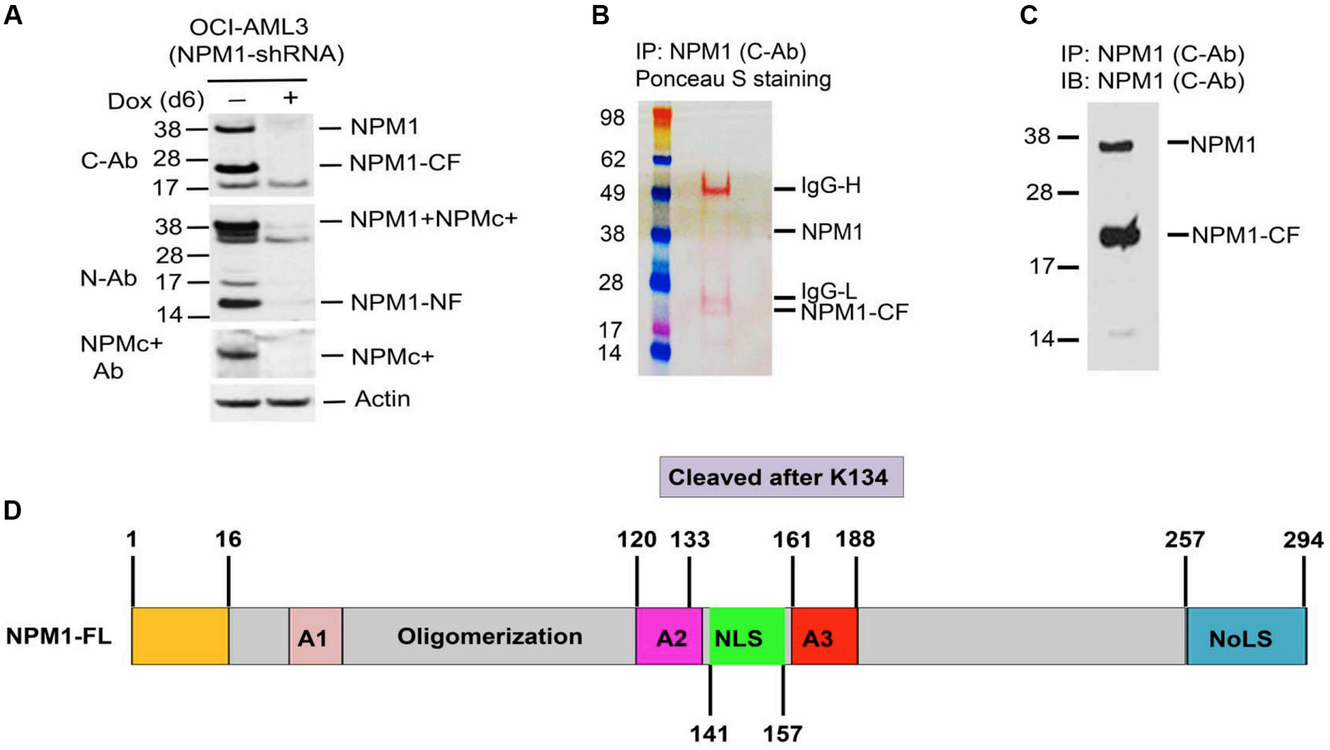

Supplementary Figure S1: Proteolytic degradation of NPM1 and identification of NPM1 cleavage site. (**A**) Validation of NPM1 antibody specificity. OCI-AML3 cells expressing an inducible shRNA to NPM1 and NPMc+ were treated for six days with doxycycline. The NPMc+ Ab specifically recognizes the C-terminus of NPMc+ alone. (**B**) Ponceau S staining and (**C**) Western blot of NPM1 immunoprecipitated from OCI-AML3 cells lysate using a murine monoclonal NPM1 antibody specific to the C-terminus of wt-NPM1. (**D**) Schematic diagram of NPM1 protein structure. The NPM1 cleavage site at K134 is located between the boundary of the second acidic domain (A2) and the nuclear localization domain (NLS), resulting in a N-terminal NPM1 fragment that contains the oligomerization domain and a C-terminal fragment that retains the NLS and the third acidic domain (A3).

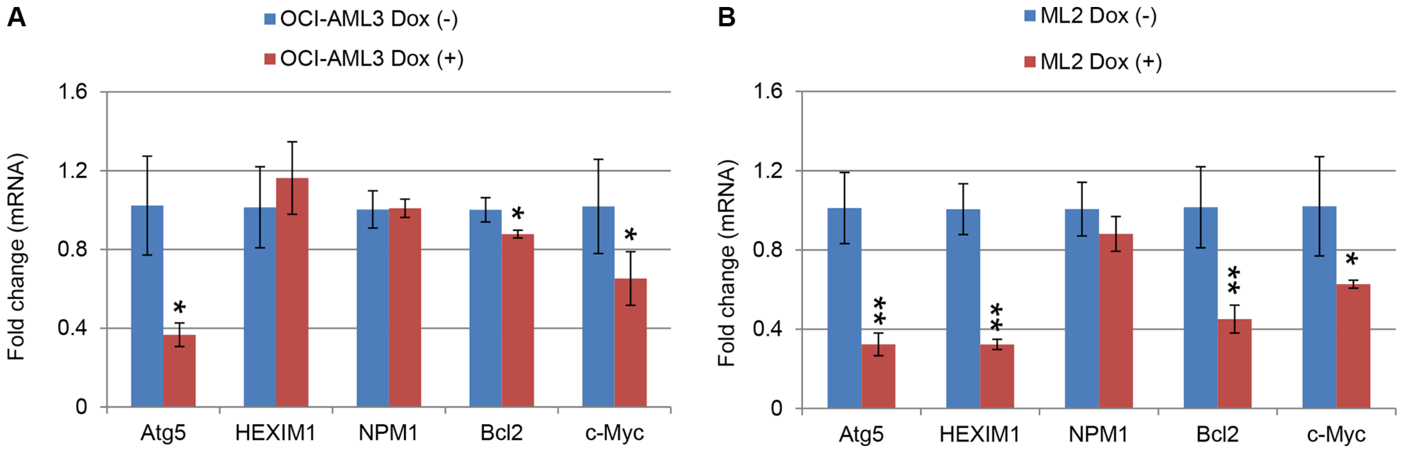

Supplementary Figure S2: Effects of Atg5 depletion on mRNA expression of NPM1/NPMc+, HEXIM1, Bcl2, and c-Myc in OCI-AML3 and ML2 cells. OCI-AML3 (**A**) and ML2 cells (**B**) stably expressing inducible Atg5-shRNA3 were incubated with doxycycline for 6 days, followed by q-PCR analysis of the mRNA levels shown. Graphs represent the mean ± S.D. of four replicates. Asterisks (*) and (**) indicate $p < 0.05$ and $p < 0.01$, respectively, in relation to control cells.

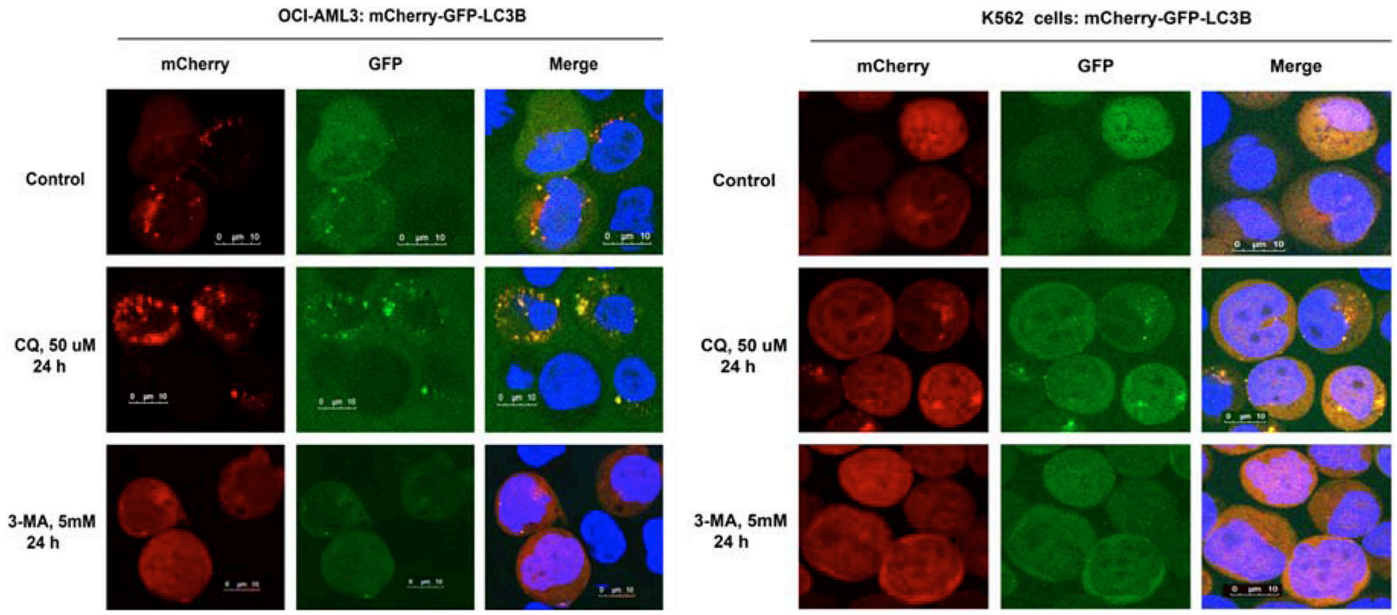

Supplementary Figure S3: Confocal microscopic images of OCI-AML3 and K562 cells expressing mCherry and GFP dual-tagged LC3B. Autophagic punctae are prevalent in OCI-AML3 cells, but not in K562 cells stably expressing mCherry and GFP dual-tagged LC3B. Scale bar represents 10 µm. Cells were incubated with the autophagy inhibitors chloroquine (CQ, 50 µM) or 3-MA (5 mM) for 24 h, fixed and stained with DAPI as described in the Materials and Methods, followed by confocal imaging analysis.

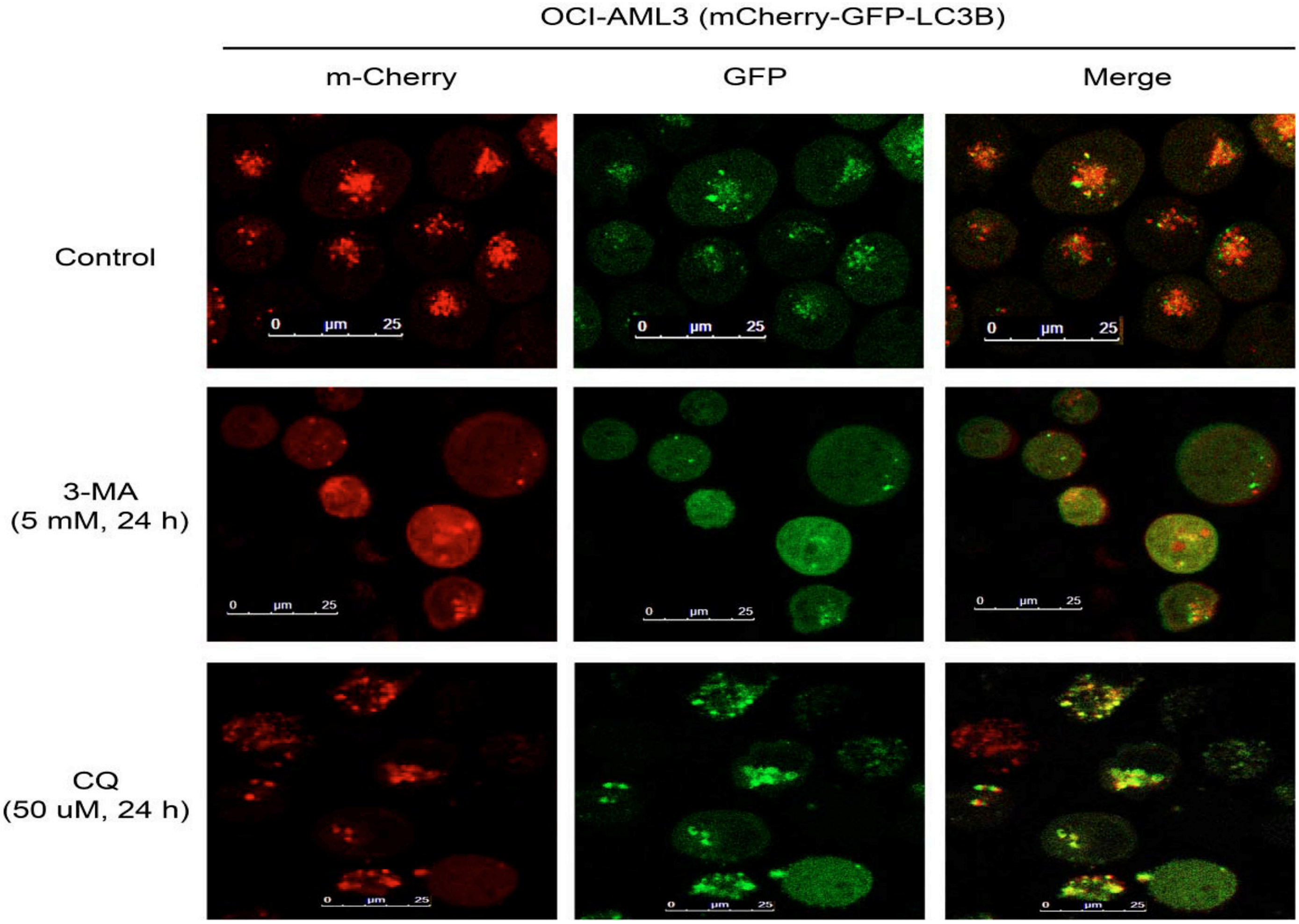

Supplementary Figure S4: Confocal microscopic live imaging of OCI-AML3 expressing mCherry and GFP dual-tagged LC3B. Cells were incubated with the autophagy inhibitors 3-MA (5 mM) or CQ (50 μM) for 24 h, followed by confocal live imaging analysis. Scale bar represents 25 μm.

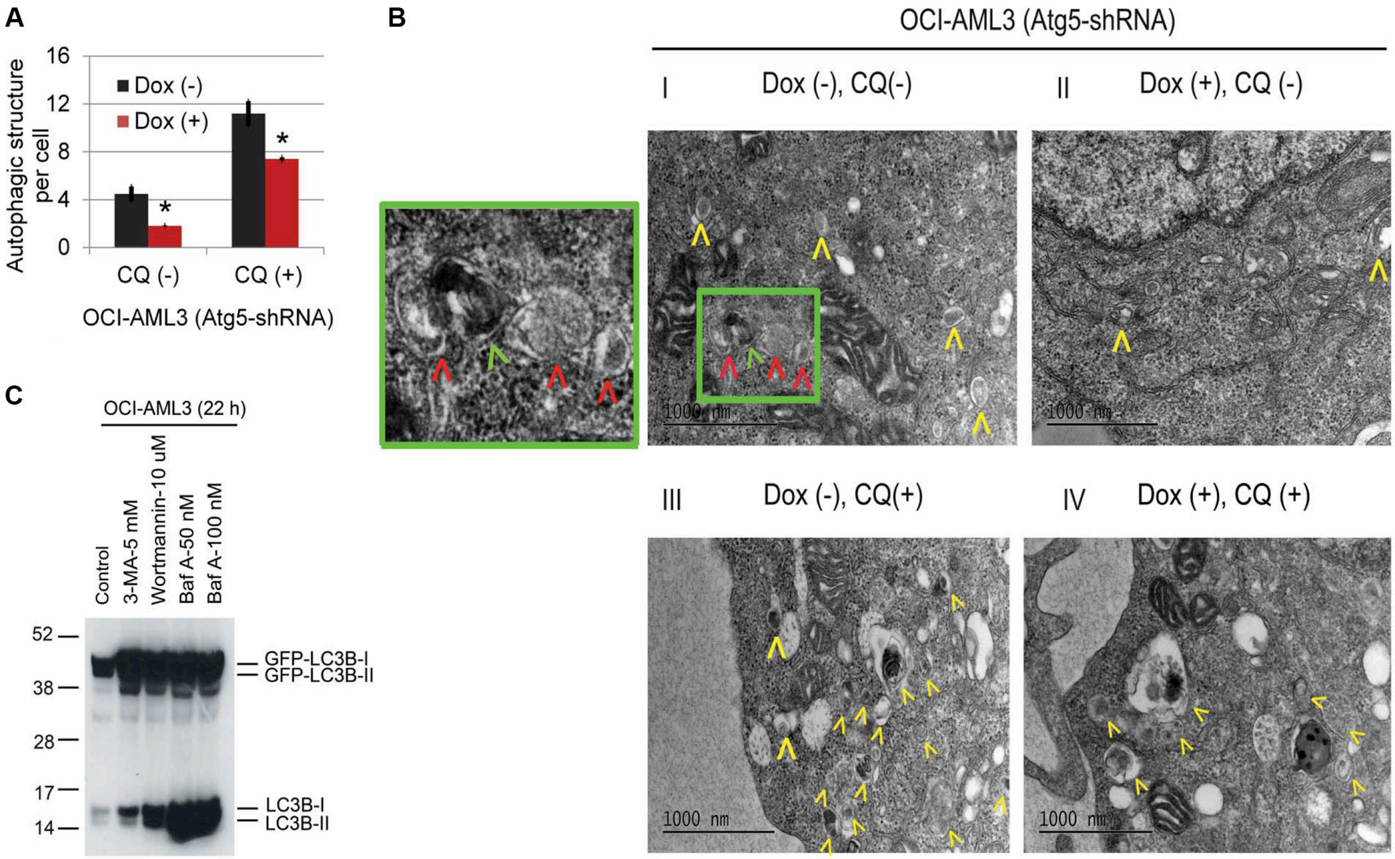

Supplementary Figure S5: Transmission electron microscopic images of autophagy structures and Western blot analysis of LC3B protein. (**A**) Quantitative determination of autophagic structures per cell. Fifty cell profiles (presence of nuclei required) obtained from the electron microscopy images were randomly selected per quadrant and autophagosome and autolysosome structures were counted manually. Experiments were performed in triplicate for each condition. The analysis was blinded to the sample at the time of quantification. Graphs represent the mean ± S.D. of biological triplicates. Asterisks (*) indicates $p < 0.05$ in relation to control cells without doxycycline induction. (**B**) Representative electron microscopic images of OCI-AML3 cells. Autophagosomes characterized by double membranes are shown as (^). (Panel 1) autophagosomes or autolysosomes in control OCI-AML3 cells. Area of magnification (insert) shown in green box; (Panel 2) Reduction in autophagosomes in OCI-AML3 cells expressing Atg5 shRNA; (Panel 3) Increase in autophagosomes after treatment with CQ (50 μM) for 24 h; (Panel 4) Effect of Atg5 knockdown in the presence of CQ. Original magnification, 8,000X. Scale bar, 1.0 μm. (**C**) OCI-AML3 cells stably expressing GFP-LC3B were incubated with the autophagy inhibitors 3-MA, Wortmannin, or BafA1 at the concentrations shown for 22 h followed by Western blot analysis (longer exposure of Figure 2F).

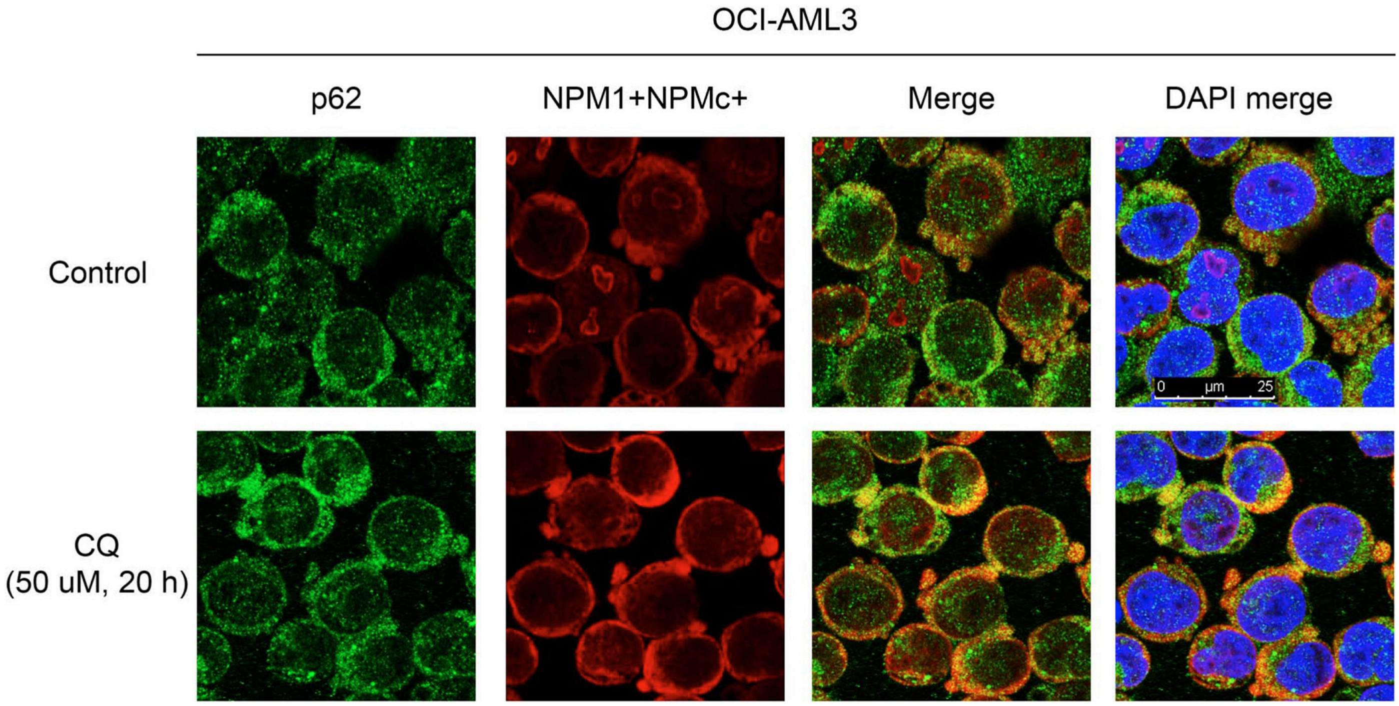

Supplementary Figure S6: Co-localization of NPM1 with p62 in OCI-AML3 cells. (**A, B**) Representative confocal images of OCI-AML3 cells, untreated or treated with CQ (50 µM) for 20 h. NPM1 and NPMc+ (red), p62 (green), and DAPI (blue). Scale bar, 25 µm

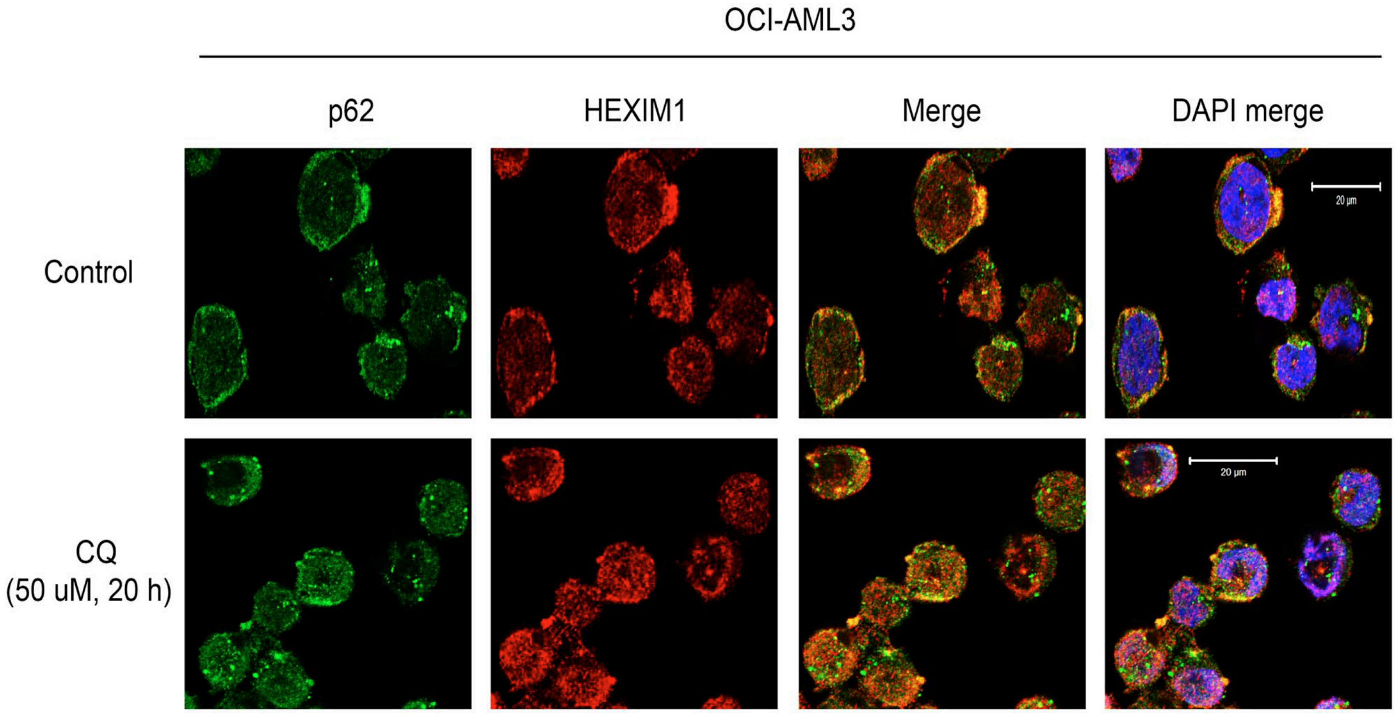

Supplementary Figure S7: Co-localization of HEXIM1 with p62 in OCI-AML3 cells. (**A, B**) Representative confocal images of OCI-AML3 cells, untreated or treated with CQ (50 µM) for 20 h. p62 (green), HEXIM1 (red), and DAPI (blue). Scale bar, 20 µm.

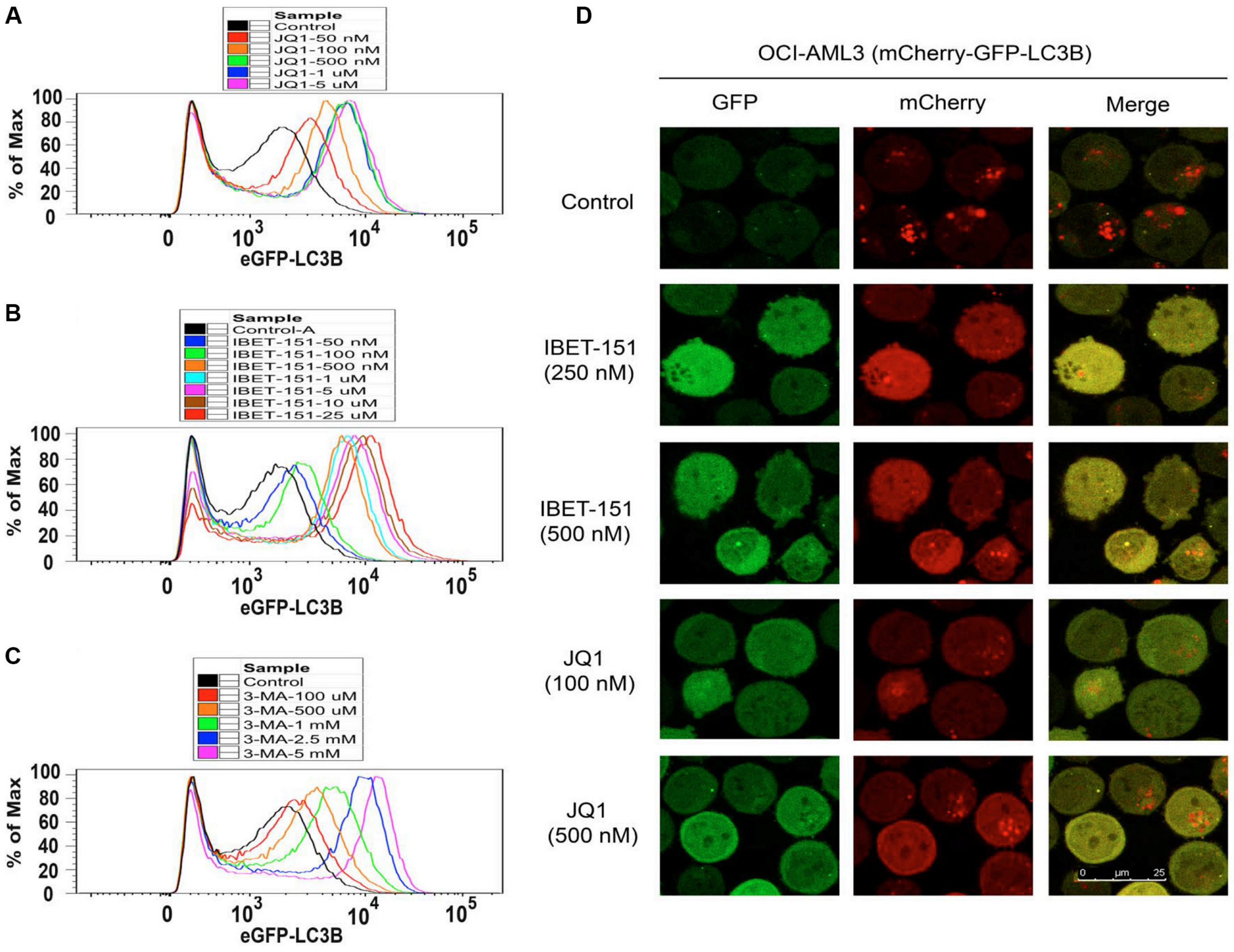

Supplementary Figure S8: Effects of BET inhibitors on GFP-LC3B fluorescence intensity and autophagy flux in OCI-AML3 cells. (**A, B, C**) Histograms of OCI-AML3 cells stably expressing eGFP-LC3B untreated or treated with the indicated concentrations of JQ1 (A), I-BET151 (B), or (-)-JQ1 for 24 h, followed by flow cytometric analysis of GFP-LC3B fluorescence intensity in OCI-AML3 cells expressing the GFP-LC3B fusion protein. (**D**) OCI-AML3 cells expressing mCherry and GFP dual-tagged LC3B were incubated with the BET inhibitors JQ1 or I-BET151 at concentrations indicated for 20 h, followed by confocal microscopic live cell imaging analysis. Scale bar represents 25 μm.

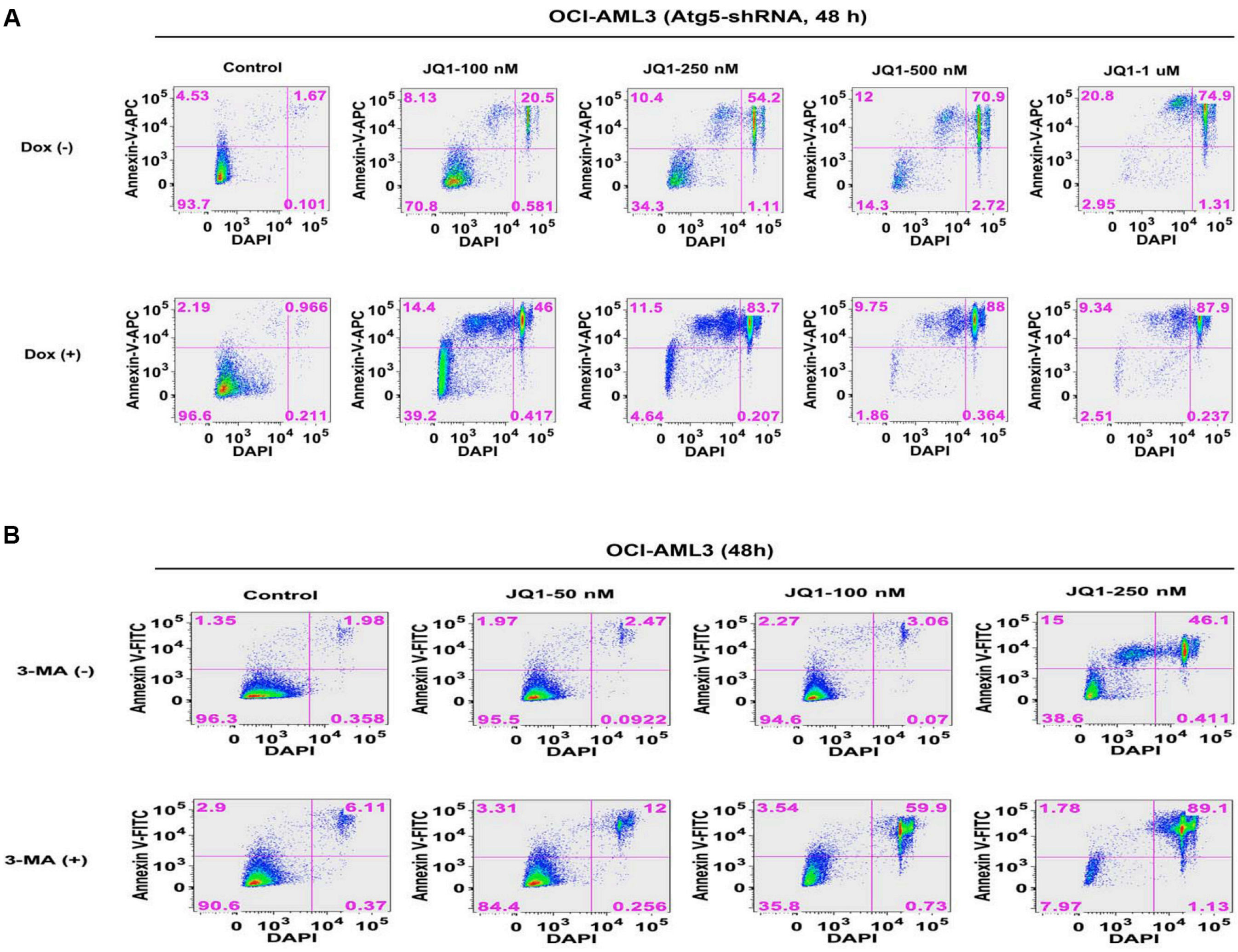

Supplementary Figure S9: Effects of autophagy inhibition on JQ-1-induced apoptosis in OCI-AML3 cells. (**A**) OCI-AML3 cells expressing inducible Atg5 shRNA3 were treated with JQ1 at the indicated concentrations for 48 h, followed by flow cytometric analysis of apoptosis as shown in representative histograms. (**B**) OCI-AML3 cells were treated with 3-MA (5 mM) or JQ1 alone or in combination at the indicated concentrations for 48 h, followed by flow cytometric analysis of apoptosis.

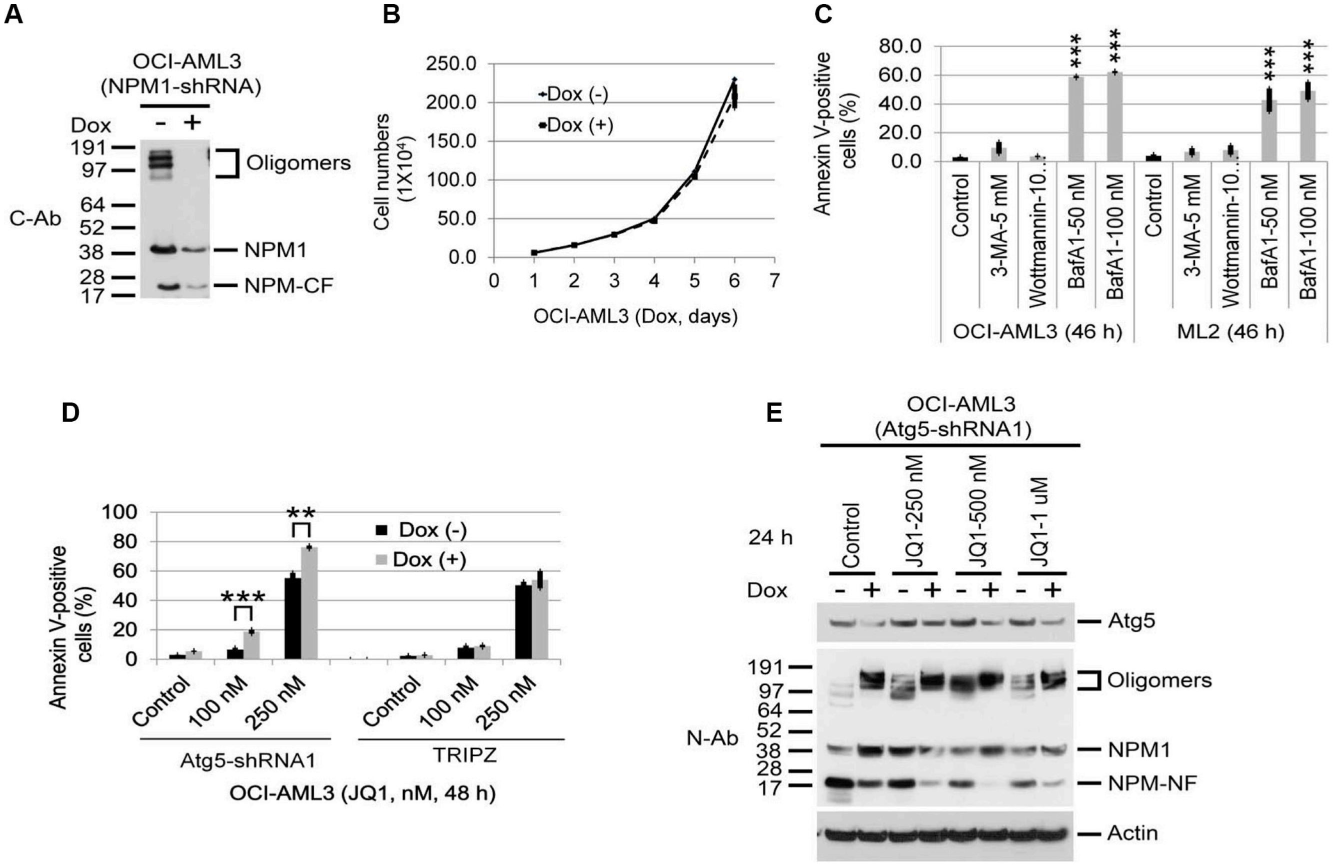

Supplementary Figure S10: Effects of autophagy inhibition on cell growth, NPM protein stability, and Annexin V positivity in OCI-AML3 and ML2 cell lines. (**A**) Validation of NPM1 antibody specificities for NPM1 oligomers. OCI-AML3 cells expressing an inducible shRNA to NPM1 and NPMc+ were treated for six days with doxycycline. C-Ab recognizes the C-terminus of wt-NPM1. (**B**) Effects of Atg5 depletion on cell growth of OCI-AML3 cells. OCI-AML3 cells stably expressing Atg5 shRNA3 were incubated with doxycycline for the days indicated. Data points represent mean values ± SD of biological triplicates. (**C**) OCI-AML3 and ML2 cells were treated with the autophagy inhibitors 3-MA, Wortmannin, or BafA1 at the concentrations shown for 46 h followed by flow cytometric analysis of Annexin V positivity. Bar graphs represent mean values ± S.D. of biological triplicate. Asterisks (***) indicates $p < 0.001$ in relation to un-treated control cells. (**D**) Effects of Atg5 depletion on JQ1-induced apoptosis in OCI-AML3 cells. OCI-AML3 cells stably expressing Atg5 shRNA1 or TRIPZ control lentiviral vector were incubated with doxycycline for five days to induce Atg5 shRNA1 expression, then treated with JQ1 for 48 h, and analyzed for Annexin V positivity by flow cytometry. The bar graphs represent mean values ± S.D. of biological triplicates. (**E**) OCI-AML3 cells stably expressing Atg5shRNA1 were incubated with doxycycline for five days to induce Atg5 shRNA1 expression, then treated with JQ1 at the indicated concentrations for 24 h, followed by Western blot analysis of the proteins shown.

Primary NPMc+ AML profiles

AML Patient ID	Age	Disease Status	Karyotype	NPM1 status	FLT3 mutation status	IDH1/2 mutation status	DNMT3A mutation status
BM1	71	Relapsed	NK	NPMc+	FLT3-ITD	IDH2 mut (p.R140Q)	No
BM5	26	Relapsed	NK	NPMc+	WT	No	No
BM7	40	Relapsed	CK	NPMc+	FLT3-D835	No	No
BM8	68	Relapsed	NK	NPMc+	FLT3-ITD	No	DNMT3A mut (p.Arg882His)
BM41	63	Relapsed	NK	NPMc+	FLT3-D835	No	No
SU302	58	De novo	NK	NPMc+	WT	No	No
SU320	68	De novo	NK	NPMc+	FLT3-ITD	IDH2 mut (p.R140Q)	No
SU484	71	De novo	NK	NPMc+	WT	No	No
SU532	54	De novo	NK	NPMc+	FLT3-ITD	No	No
SU575	72	De novo	NK	NPMc+	FLT3-ITD	No	DNMT3A mut (p.Arg882His)
SU623	64	De Novo	NK	NPMc+	FLT3-ITD	No	No

Supplementary Figure S11: Characterization of mutations in primary AML cells was performed in a CLIA-approved laboratory and the genotypes for each primary cell sample are shown.

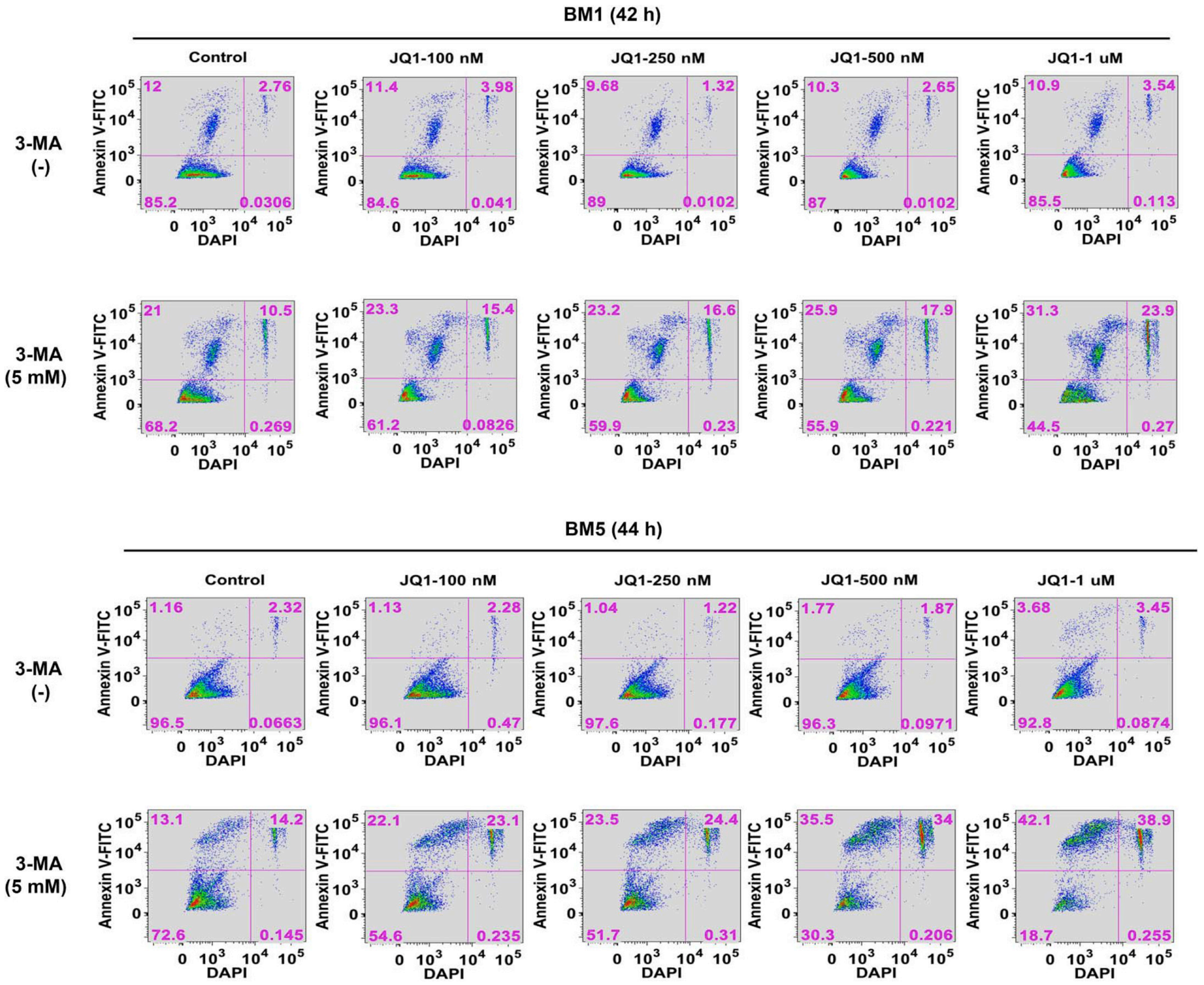

Supplementary Figure S12: Effects of 3-MA on JQ1-induced apoptosis in primary NPMc+ AML cells. Primary NPMc+ AML cells from BM1 and BM5 were treated with 3-MA or JQ1 alone or in combination at the indicated concentrations and times, followed by flow cytometric analysis of apoptosis as shown in representative histograms.

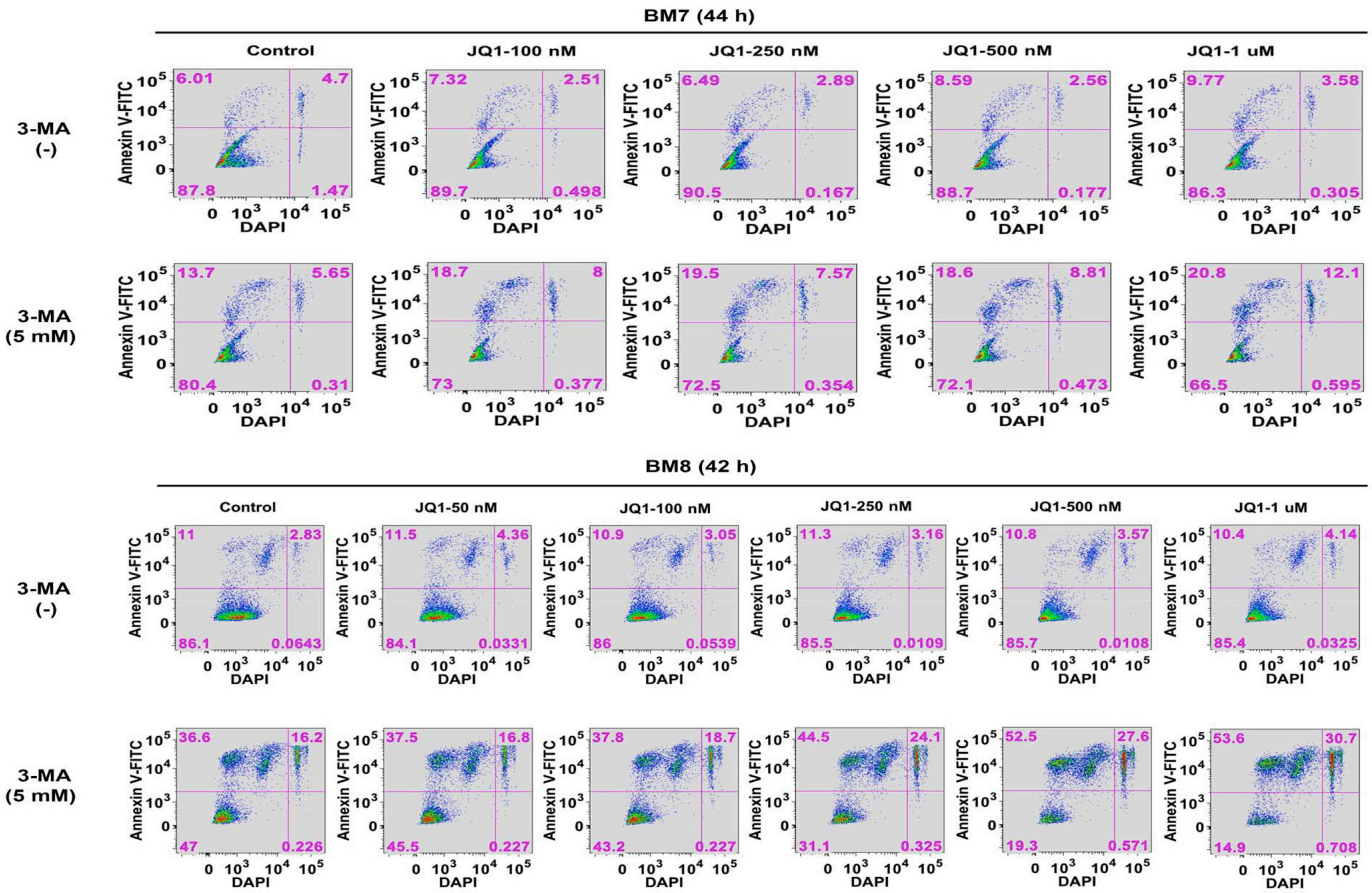

Supplementary Figure S13: Effects of 3-MA on JQ1-induced apoptosis in primary NPMc+ AML cells. Primary NPMc+ AML cells from BM7 and BM8 were treated with 3-MA or JQ1 alone or in combination at the indicated concentrations and times, followed by flow cytometric analysis of apoptosis as shown in representative histograms.

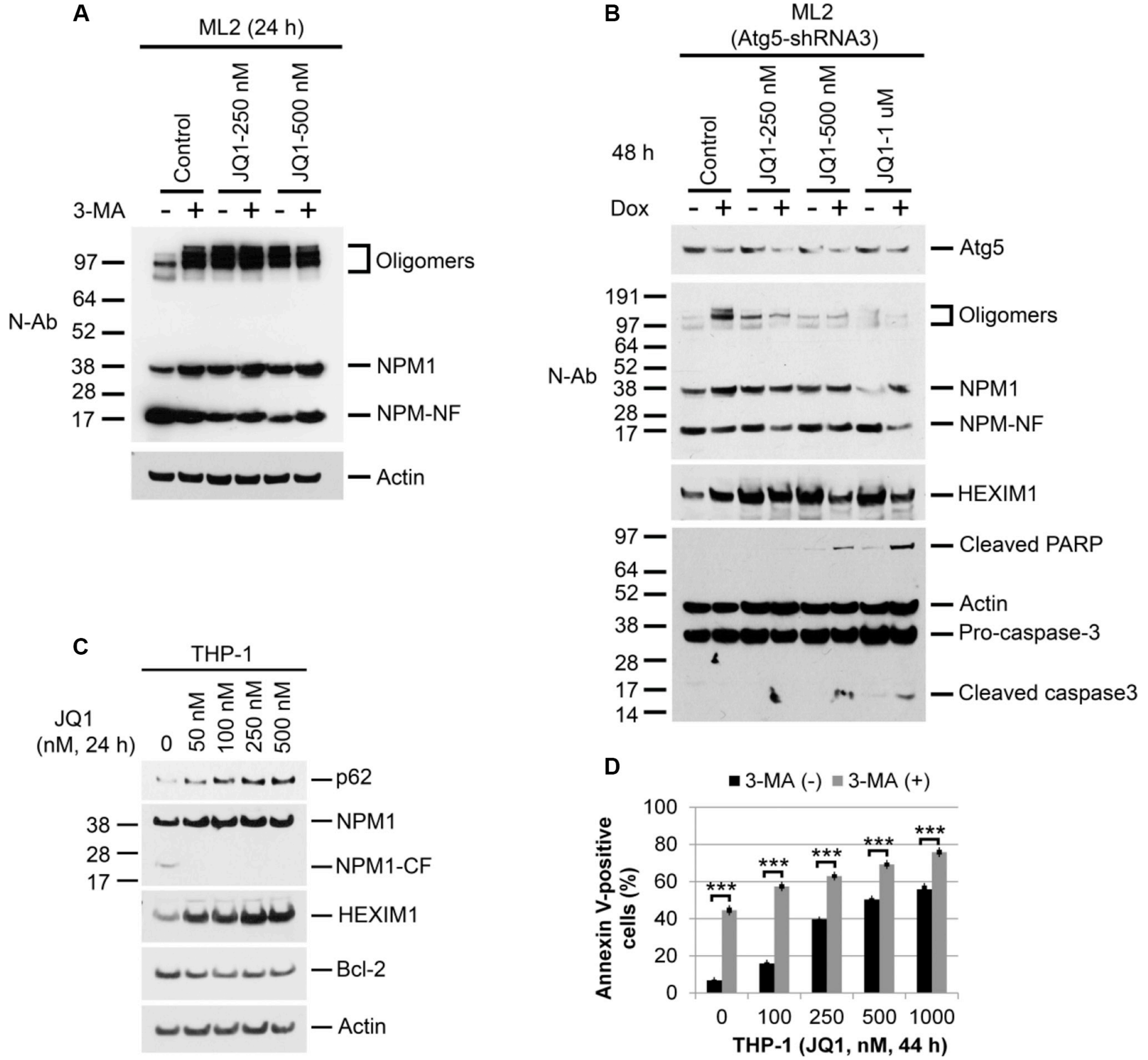

Supplementary Figure S14: Effects of BET and autophagy inhibition on protein expression and Annexin V positivity in MLL cell lines. (**A**) ML2 cells were treated with JQ1 or 3-MA (5 mM) alone or in combination at the concentrations shown for 24 h, followed by Western Blot analysis of the proteins shown. (**B**) Effects of Atg5 depletion and JQ1 on protein expression in ML2 cells. ML2 cells stably expressing Atg5 shRNA3 were incubated with doxycycline for six days, then treated with JQ1 at the indicated concentrations for 48 h followed by Western blot analysis of the proteins shown. (**C**) THP-1 cells were treated with JQ1 at the indicated concentrations for 24 h, followed by Western blot analysis of the proteins shown. (**D**) THP-1 cells were treated with JQ1 or 3-MA (5 mM) alone or in combination at the indicated concentrations and times, followed by flow cytometric analysis of Annexin V positivity. Bar graphs represent mean values ± SD of biological triplicates. Asterisks (***) indicate $p < 0.001$ in relation to the corresponding control cells without doxycycline induction.

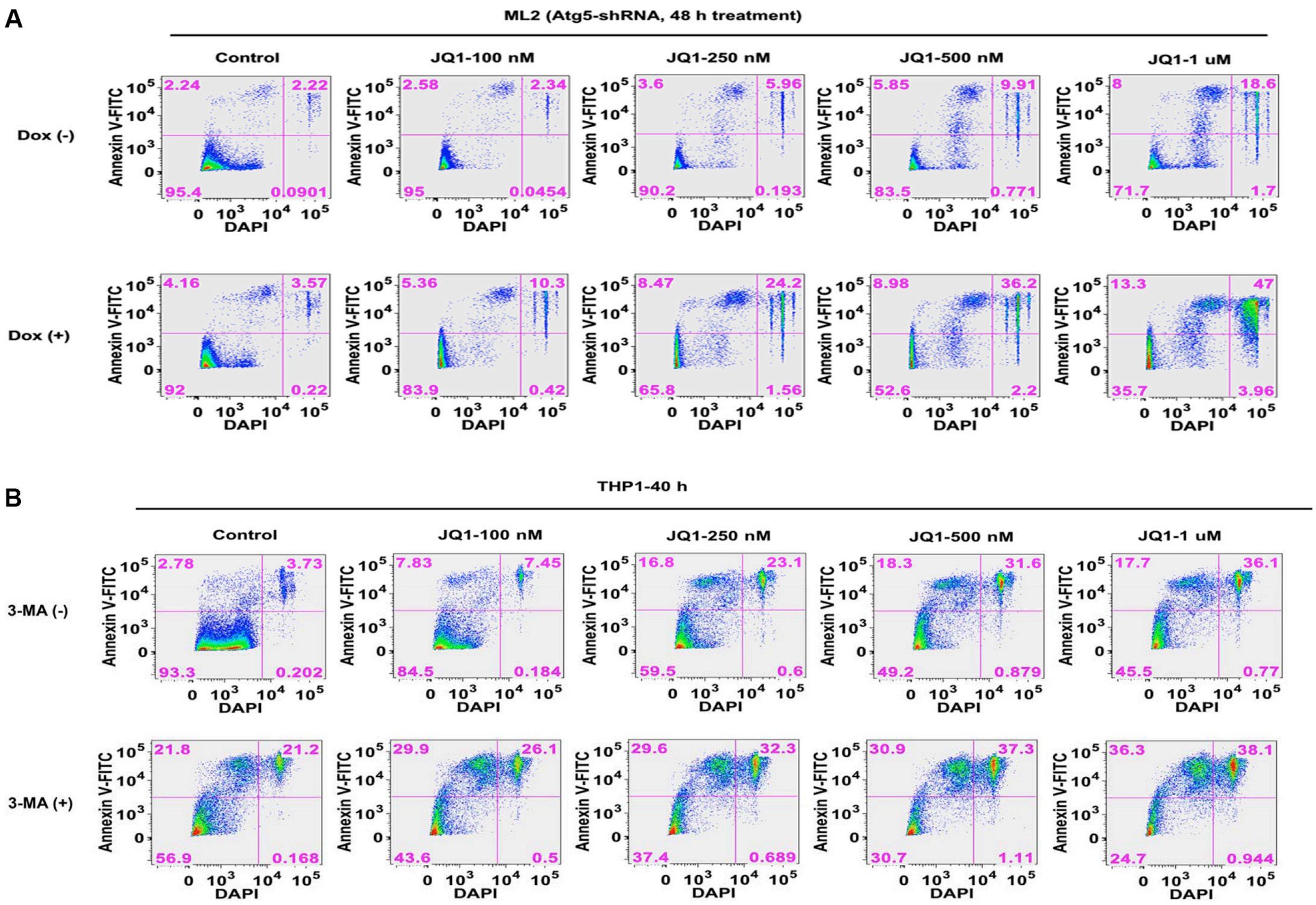

Supplementary Figure S15: Effects of autophagy inhibition on JQ1-induced apoptosis in ML2 and THP-1 cells. (**A**) ML2 cells expressing inducible Atg5 shRNA were treated with JQ1 at the indicated concentrations for 48 h, followed by flow cytometric analysis of apoptosis as shown. (**B**) THP-1 cells were treated with 3-MA (5 mM) or JQ1 alone or in combination at the indicated concentrations for 40 h, followed by flow cytometric analysis of apoptosis as shown.

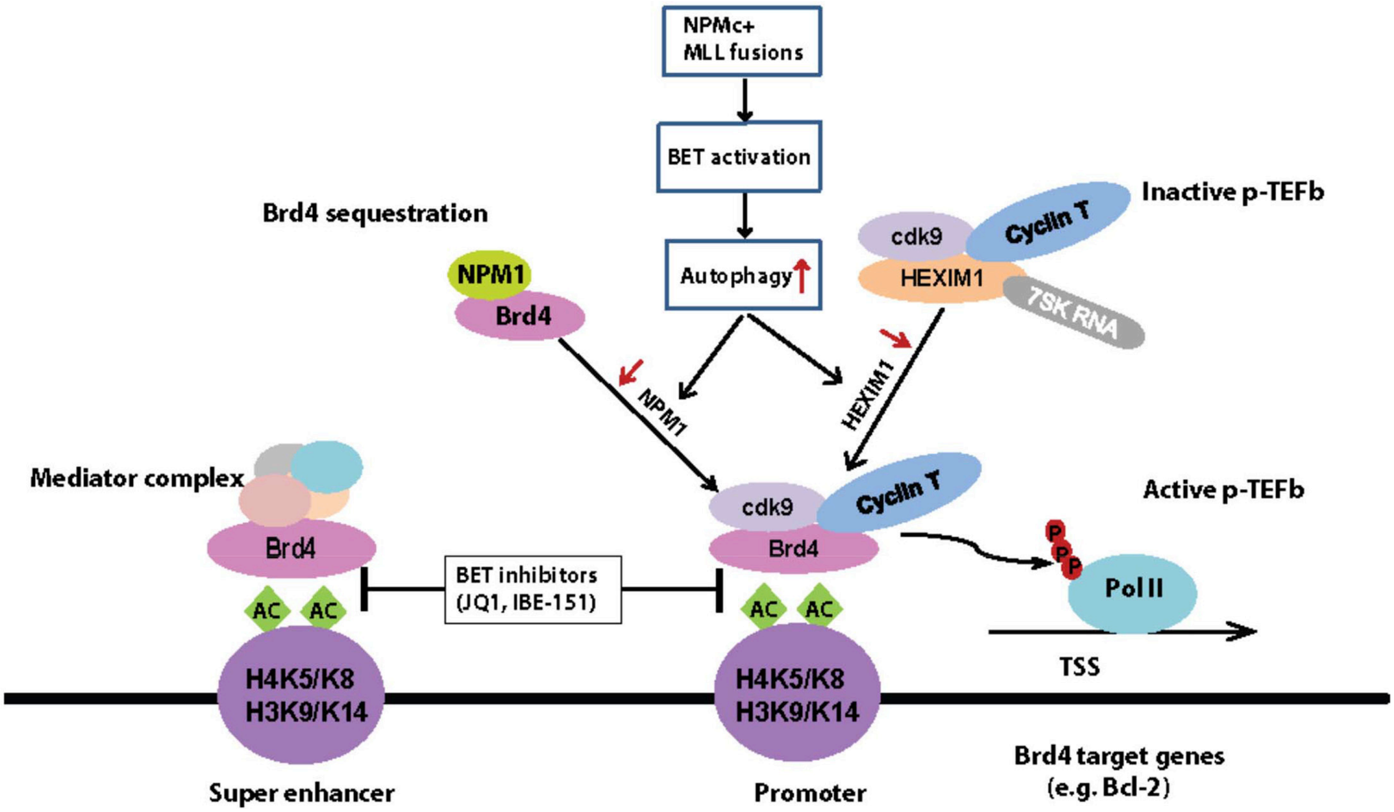

Supplementary Figure S16: Schema illustrating the proposed interaction between autophagy and BET activation. Acetylated histones recruit the positive transcription elongation factor B (pTEFb), the complex formed by cyclin-dependent kinase 9 (CDK9) and its activator cyclin T1 to promoter regions, resulting in the phosphorylation of the carboxy-terminal heptat repeat region of RNA polymerase II (RNA Pol II) and transcriptional elongation. Brd4 interacts with the pTEFb complex at super-enhancer regions to facilitate this process. Brd4 is enriched at promoter and super-enhancer regions and strongly stimulates the expression of Brd4 target genes that include Bcl-2 and c-Myc. Small-molecule bromodomain inhibitors (JQ1 or I-BET151) that bind Brd4 repress the expression of Brd4 transcriptional targets. NPM1 and HEXIM1 play a negative role in p-TEFb activation by reducing the binding of Brd4 to p-TEFb. Reduced expression of NPM1 and HEXIM1 through autophagy diminishes their inhibitory effects on the p-TEFb complex and increases the expression of Brd4 target genes, whereas BET inhibitors inhibit autophagy, increase HEXIM1 and NPM1 expression, and decrease BET-dependent gene expression. TSS, transcription start site.

Supplemental Table S1: Primers sequences used for real time-RT-PCR analysis

Gene	Primer	Primer sequences	Amplicon size (bp)	Genebank Accession #
NPM1	Forward Reverse	5′-TTGTTGAAGCAGAGGCAATG-3′<br>5′-AATATGCACTGGCCCTGAAC-3′	158	NM_002520.6
Bcl2	Forward Reverse	5′-CTTTGAGTTCGGTGGGGTCA-3′<br>5′-GGGCCGTACAGTTCCACAAA-3′	162	NM_000633.2
HEXIM1	Forward Reverse	5′-AAGGACTAGCTAAAGGCGTCAC-3′<br>5′-TGGCTAGTAGAGTCCTCGAAGTTT-3′	155	NM_006460.2
GAPDH	Forward Reverse	5′-CCCCTTCATTGACCTCAACTACAT-3′<br>5′-CGCTCCTGGAAGATGGTGA-3′	135	NM_002046
